# Common Patterns of Hydrolysis Initiation in P-loop Fold Nucleoside Triphosphatases

**DOI:** 10.3390/biom12101345

**Published:** 2022-09-22

**Authors:** Maria I. Kozlova, Daria N. Shalaeva, Daria V. Dibrova, Armen Y. Mulkidjanian

**Affiliations:** 1 School of Physics, Osnabrueck University, D-49069 Osnabrueck, Germany; 2 Center of Cellular Nanoanalytics, Osnabrueck University, D-49069 Osnabrueck, Germany

**Keywords:** Walker ATPase, Walker A motif, Walker B motif, ATPase, Ras GTPase, arginine finger, lysine finger, ABC transporter, myosin, kinesin, aluminum fluoride, magnesium fluoride

## Abstract

The P-loop fold nucleoside triphosphate (NTP) hydrolases (also known as Walker NTPases) function as ATPases, GTPases, and ATP synthases, are often of medical importance, and represent one of the largest and evolutionarily oldest families of enzymes. There is still no consensus on their catalytic mechanism. To clarify this, we performed the first comparative structural analysis of more than 3100 structures of P-loop NTPases that contain bound substrate Mg-NTPs or their analogues. We proceeded on the assumption that structural features common to these P-loop NTPases may be essential for catalysis. Our results are presented in two articles. Here, in the first, we consider the structural elements that stimulate hydrolysis. Upon interaction of P-loop NTPases with their cognate activating partners (RNA/DNA/protein domains), specific stimulatory moieties, usually Arg or Lys residues, are inserted into the catalytic site and initiate the cleavage of gamma phosphate. By analyzing a plethora of structures, we found that the only shared feature was the mechanistic interaction of stimulators with the oxygen atoms of gamma-phosphate group, capable of causing its rotation. One of the oxygen atoms of gamma phosphate coordinates the cofactor Mg ion. The rotation must pull this oxygen atom away from the Mg ion. This rearrangement should affect the properties of the other Mg ligands and may initiate hydrolysis according to the mechanism elaborated in the second article.

## 1. Introduction

Hydrolysis of nucleoside triphosphates (NTPs), such as ATP or GTP, by so-called P-loop fold nucleoside triphosphatases (also known as Walker NTPases) is one of the key enzymatic reactions. P-loop NTPase domains drive the activity of rotary ATP synthases, DNA and RNA helicases, kinesins and myosins, ABC-transporters, and most GTPases, including ubiquitous translation factors, as well as α-subunits of heterotrimeric G-proteins [1,2,3,4,5,6,7,8,9,10,11,12,13,14,15]. Furthermore, human Ras GTPases, involved in many types of cancer, get converted into active oncoproteins by mutations in positions 12, 13, or 61 of the GTP-binding pocket [16,17,18].

In the ECOD database [19], the topology-level entry “P-loop_NTPase” contains 193 protein families. In the Pfam database [20], the P-loop NTPase clan CL0023 contains 217 families. The main classes of P-loop NTPases were already present in the last universal cellular ancestor (LUCA) [7,8,13,15,21,22,23,24,25].

Although up to 20% of the genes in a typical cell encode different P-loop NTPases, there is no consensus on the hydrolysis mechanism. At the same time, P-loop NTPases belong to the enzyme families with the best sampling of structures such that comparative structure analysis can be used to clarify their catalytic mechanism, see e.g., [4].

**Figure 1:**
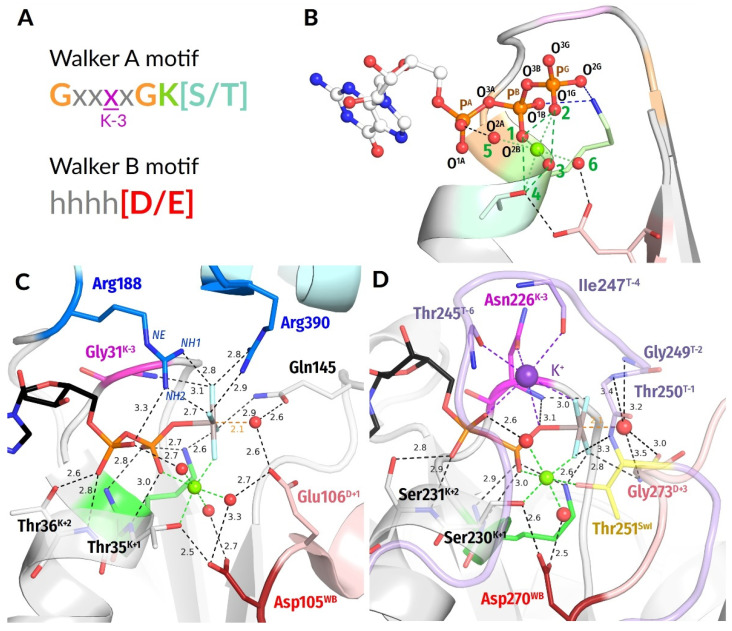
**P-loop-fold NTPases.** (**A**) Conserved motifs in P-loop NTPases; (**B**) naming of atoms according to IUPAC recommendations for nucleoside triphosphates [27], and typical Mg^2+^ coordination, where the green numbers denote the six ligands; (**C**,**D**) crystal structures of typical catalytic sites of P-loop NTPases. (**C**) Superfamily 1 helicase Pif1 with a transition state analogue ADP-AlF_4_^−^ bound (PDB ID 5FHD, [28]). (**D**) K^+^-dependent GTPase MnmE with a transition state analogue GDP-AlF_4_^−^ bound (PDB ID 2GJ8 [29]). Colour code: polypeptide chains of P-loop domains are shown as gray cartoons; other than P-loop domain subunits carrying Arg/Lys fingers are shown in cyan; nucleotides, their analogues, and important amino acid residues are shown as sticks; water molecules as red spheres; Mg^2+^ ions as lime spheres, the K^+^ ion as a purple sphere; the conserved Lys residue of Walker A motif (K^WA^) is shown in green, and the residue three positions before it (K−3) is highlighted in magenta; the conserved Asp residue of Walker B motif is shown in dark red; and the Arg fingers are shown in blue. In those amino acid residues that are shown as sticks, the oxygen atoms are colored red, and the nitrogen atoms are colored blue. In the AlF_4_^−^ moiety, the Al atom is colored gray, and the fluoride atoms are colored light blue. The switch I/K-loop motif is shown in lilac, and the conserved Thr^SwI^ is highlighted in yellow.

In P-loop NTPases, the eponymous P-loop, together with the first two residues of the following α-helix, usually follows the sequence pattern [G/A]xxxxGK[S/T], known as the Walker A motif [1]. This motif stabilises the triphosphate chain of NTP and the cofactor Mg^2+^ ion [1,2,26], see (Figure 1A–D). The Walker B motif hhhh[D/E], where “h” denotes a hydrophobic residue, is the other common motif of P-loop NTPases that is found on the C-terminal tip of that β-strand, which is opposite the Walker A α-helix, (Figure 1A,B) [1,2,26].

A specific feature of most P-loop ATPases is their activation before each turnover—otherwise, uncontrolled NTP hydrolysis would be perilous for cell survival. Usually, an ATP- or GTP-bound P-loop domain interacts with its cognate activating partner, which could be a domain of the same protein, a separate protein, and/or a DNA/RNA molecule. Upon this interaction, specific stimulatory moieties, usually Arg or Lys residues (“Arg/Lys finger(s)” Figure 1C), are inserted into the catalytic site and initiate the cleavage of γ-phosphate [4,12,29,30,31,32,33,34,35,36,37,38].

Mutations of a conserved lysine residue in the Walker A motif, aspartate in the Walker B motif, or stimulatory residues drastically reduce enzyme activity unless the substituents are amino acids with very similar properties (Glu instead of Asp or Lys instead of Arg). In the latter case, some activity may be retained [36].

Important hints for clarifying the events inside the catalytic sites of P-loop NTPases are provided by their structures with bound transition state (TS) analogues, such as NDP:AlF_4_^−^ or NDP:MgF_3_^−^ or NDP-VO_4_^3−^ complexes [4,30,31,37,39,40,41,42,43,44]. It is anticipated that these analogues promote the formation of a fully fledged active site in a precatalytic state. In such complexes, a water molecule is often located apically to the analogue of γ-phosphate, which corroborates the earlier suggestions that γ-phosphate cleavage is triggered by the apical nucleophilic attack of a polarized water molecule (W_cat_) after its deprotonation to OH^—^_cat_ [12,37,43,44,45,46,47,48,49], see (Figure 1C,D). In addition, TS analogues have been shown to promote the interaction of the activator with the NTPase domain; therefore, they often disclose the interaction of the stimulator with the triphosphate chain, as shown in Figure 1C,D and discussed in [30,31,37,38,39,40,41,43,50,51,52,53,54,55,56,57,58,59].

There is no consensus on how stimulators initiate the hydrolysis. Warshel and colleagues suggested that stimulators, owing to their positive charge, compensate the negative charges of oxygen atoms of triphosphate and make the γ-phosphorus atom (P^G^) more prone to the nucleophilic attack by a water molecule [60,61,62]. It was also suggested that the pushing of bound water molecules out of the binding pocket into the bulk solution by the stimulatory arginine finger provides an entropic gain [63]. Jin and colleagues proposed reorientation of the W_cat_ molecule into the attack position by the stimulator [43,64,65]. In a later reaction step, stimulators were implied in compensating for the negative charge that develops at β-phosphate upon the breakaway of γ-phosphate [49,66]. Recently, Gerwert and colleagues proposed, for small GTPases, that the stimulatory Arg finger rotates the α-phosphate group towards an eclipsed conformation with respect to β- and γ-phosphates, which would destabilize the triphosphate chain [67,68,69].

Elsewhere [70], we have focused on P-loop NTPases that are stimulated not by Arg/Lys residues, but by K^+^ and Na^+^ ions [29,35,71,72,73,74]. We performed molecular dynamics (MD) simulations of the Mg^2+^-GTP-containing dimer of the K^+^-dependent tRNA-modifying enzyme MnmE (see Figure 1D) and compared the results with MD simulations of water-dissolved Mg^2+^-GTP and Mg^2+^-ATP complexes in the presence of monovalent cations. In the case of water-dissolved NTPs, one of the monovalent cations bound between the O^2A^ and O^3G^ atoms of α- and γ-phosphates, in the so-called AG site, which was accompanied by rotation of α-phosphate—as the least constrained phosphate group—yielding a fully eclipsed configuration of the triphosphate chain [70]. These data agreed with the results of Gerwert and colleagues, who used a Mg^2+^-methyltriphosphate complex—as a simple mimic of Mg^2+^-GTP—in their modeling of the interaction with an Arg finger [67].

However, MD simulations of the GTP-containing MnmE protein dimer showed that α-phosphate was fully immobilized by bonds between the GTP molecule and the residues of the P-loop: these interactions stabilized the triphosphate chain in an extended, supposedly catalytically prone conformation, as seen in Figure 1D. In this case, only the terminal γ-phosphate retained some mobility. The K^+^ ion, by binding between the O^2A^ and O^3G^ atoms of the protein-bound GTP molecule, caused twisting of the γ-phosphate group by approximately 30°; the rotation was counterclockwise when viewed from the side of γ-phosphate (Figure S2) [70]. It is important that the K^+^ ion can hardly link these two oxygen atoms without twisting γ-phosphate. The rotated γ-phosphate was stabilized by a new H-bond between the O^2G^ atom of γ-phosphate and the backbone amide group three residues before the conserved lysine residue of Walker A motif (K-3 residue in Figure 1A–D and Figure S2). Both the twisting of γ-phosphate and its stabilization by an additional H-bond could potentially promote hydrolysis [70]. Still, these features have not been discussed in relation to the catalytic mechanisms of any P-loop NTPase before. Therefore, we were interested to find out how widespread the features we identified in K^+^-stimulated GTPase MnmE are.

Previous comparative structure analyses of P-loop NTPases considered several representative structures [4,75,76]. Here, we performed the first, to our best knowledge, comparative structure analysis of all available P-loop NTPase structures with fully fledged catalytic sites. We screened 1484 available structures of P-loop NTPases with bound Mg-NTPs or their analogues (in total, 3666 individual catalytic sites were analyzed). We proceeded on a general assumption that structural features common to these P-loop NTPases may be essential for catalysis. We also relied on a key observation that diverse P-loop NTPases, despite playing dramatically different roles in the cell, catalyze essentially the same or similar chemical reactions [9]. It can be expected that catalysis of congruent chemical reactions in related enzymes is accomplished by homologous structural elements.

Our results are presented in two articles. Here, in the first, we analyzed those features of stimulatory moieties that are shared by diverse P-loop NTPases. We found only two types of interaction between stimulatory moieties and triphosphate chains. In most cases, as in the MnmE GTPase, at least one stimulator links O^2A^ and O^3G^, which enforces a counterclockwise rotation of γ-phosphate. Otherwise, the stimulators interact only with the γ-phosphate group. Then the direction of twisting/pulling of γ-phosphate should be clarified on a case-to-case basis. In addition, we noticed that not all stimulators carry a full positive charge. In diverse classes of P-loop NTPases, we found asparagine, serine, and glycine residues forming stimulatory bonds with oxygen atoms of the triphosphate chain. In general, the only feature that seems to be common to all the studied P-loop NTPases is the mechanistic interaction of diverse stimulators with the γ-phosphate oxygen atoms.

This common feature allowed us to suggest that stimulators may initiate NTP hydrolysis by rotating γ-phosphate and displacing the O^1G^ atom that coordinates the Mg^2+^ ion. Such a rotational displacement of the negatively charged, Mg^2+^-coordinating oxygen atom may initiate hydrolysis by affecting the properties of the other Mg^2+^ ligands. In our second article (see the companion paper [77]) we look for the common structural features related to the subsequent steps of catalytic reaction, consider the functional consequences of the γ-phosphate rotation, and suggest a common catalytic mechanism for P-loop fold NTPases based on our comparative structure analysis.

## 2. Materials and Methods

**Figure 2:**
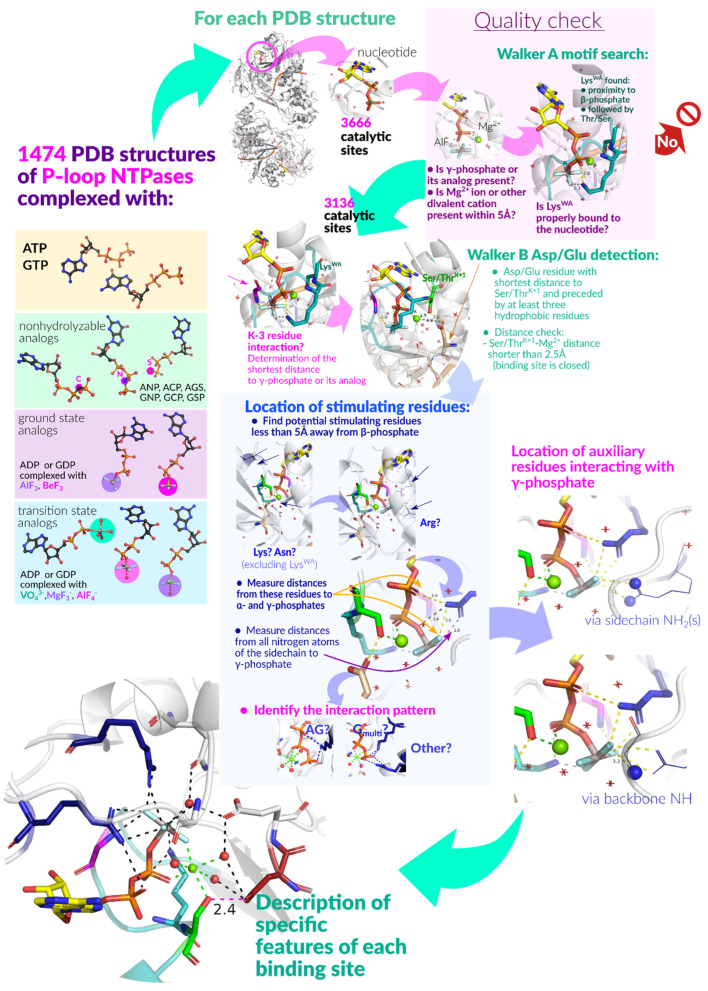
**Automated comparative structural analysis of P-loop NTPases.** The examination pipeline, as applied to the structure of each selected nucleotide-binding site, is shown. We checked for (i) the presence of the HN^K−3^—O^2G^ bond, (ii) the length of the [Asp/Glu]^WB^—[Ser/Thr]^K+1^ H-bond, and (iii) the type of interaction of stimulatory residues.

***Global computational analysis of stimulatory patterns in the structures of P-loop NTPases.*** The structures of fully fledged catalytic sites of P-loop NTPases were selected among those PDB entries that matched the following criteria: (1) the entry is assigned to the InterPro record IPR027417 “P-loop containing nucleoside triphosphate hydrolase”; (2) contains an ATP/GTP molecule, or a nonhydrolyzable analogue of NTP, or a transition-state analogue; (3) contains at least one Mg^2+^, Mn^2+^ or Ca^2+^ ion. We considered X-ray structures, as well as cryo-EM and NMR structures. This search yielded 1474 structures with 3666 catalytic sites in them. For the NMR structures, only the first frame containing an NTP analogue bound to the P-loop region was included. For X-ray and cryo-EM structures, an additional criterion was applied: structures with resolution worse than 5 Å were excluded. In the case of structures containing multiple subunits or multiple copies of the same protein, each interaction between a protein and an NTP-like molecule was treated separately, as an individual complex. The pipeline that was applied to each complex is depicted in Figure 2.

The conservation of the shape of Mg-NTP-bound P-loop as a rigid structural element enabled us to employ a mostly sequence-agnostic, distance-based approach for batch examination of catalytic sites.

To identify structures with a substrate or its analogue bound to the P-loop motif, we applied the following filter: an NTP(-like) molecule was considered bound to the P-loop if both Mg^2+^/Ca^2+^/Mn^2+^ cation and the NZ atom of Lys^WA^ can be found within 5 Å of the oxygen atoms of the β-phosphate. Furthermore, Lys^WA^ must be followed in the sequence by a Ser or Thr residue. Complexes with molecules annotated as nonhydrolyzable NTP analogues but lacking β- and/or γ-phosphate-mimicking groups were not included in the analysis.

In total, we analyzed 3136 complexes in 1383 structures with various substrates: ATP and GTP, nonhydrolyzable analogues of ATP and GTP, and ADP or GDP molecules associated with γ-phosphate-mimicking moieties (see Figure 2).

In each catalytic site under consideration, the K−3 residue of the Walker A motif (three residues before the conserved lysine residue)was identified by its position relative to the P-loop Lys residue. Distances (in Å) were measured from the backbone amide group (hereafter **HN** group) of the fourth residue of the Walker A motif to the nearest oxygen or fluorine atom of the *γ-*phosphate or its mimic. We accounted for differences in phosphate oxygen atom numeration among studied structures.

Putative [Asp/Glu]^WB^ residue was identified as follows: distances from all Asp and Glu residues to [Ser/Thr]^K+1^ were measured, and the closest residue was chosen as the partner of [Ser/Thr]^K+1^ provided that it was preceded by at least three nonionizable residues (Glu, Asp, Ser, Thr, Tyr, Lys, Arg, and His were considered as ionizable). Residues located further than 5 Å were not considered.

We also measured distances from [Ser/Thr]^K+1^ to Mg^2+^, to ensure the correct binding of the Mg^2+^ and general reliability of the structure resolution at the binding site (i.e., very long distance would indicate a disturbed catalytic site or resolution at the site that is insufficient for our purposes of comparative analysis), and from [Asp/Glu]^WB^ to Mg^2+^, to identify cases of direct coordination of Mg^2+^ by the acidic residue (too short distances) or disassembled binding sites (too long distances).

As a next step, we inspected the presence of positively charged stimulatory residues near the phosphate chains in all complexes. To identify such residues, we considered Arg and Lys residues nearest to the β-phosphate group oxygen atoms or its structural analogues (excluding the lysine of P-loop proper). Distances were then measured from the guanidinium-group nitrogen atoms (NE, NH1, NH2) of the Arg residue or from the NZ atom of the Lys residue to the closest oxygen atom of α-phosphate moiety and to the nearest fluorine or oxygen atom of γ-phosphate or its mimicking group. Similarly, we checked possible interactions of the phosphate chain with the ND2 atom of Asn. Possible additional interactions of γ-phosphate oxygens with N atoms of protein backbone and side chains in vicinity were also listed for each complex. For all these interactions, the distance threshold was 4 Å.

In the systematic analysis of all available structures, the patterns of Arg finger binding were assigned automatically, based on the composition of H-bonds between the Arg residue and the substrate molecule. We considered each pair of possible donor and acceptor atoms, where donors were NE/NH1/NH2 atoms of Arg residue and the acceptors were oxygen/fluorine atoms of the substrate. The presence of an H-bond was inferred from the atomic distance: an H-bond was stated at distances less or equal 3.2 Å, a weak H-bond stated at distances between 3.2 and 4 Å, and no possibility of an H-bond was stated for distances over 4 Å [78,79,80]).

To assign the arginine finger types, several sets of criteria were applied consecutively, as described in the Results. Structures that did not fit any of the criteria were additionally inspected and Arg binding patterns assigned manually. After all the Arg fingers were categorized, the frequency of each interaction type was determined together for the automatically and manually assigned types.

The lysine residues were assumed to be present in an AG site if both NZ-Oα and NZ-Oγ distances were shorter than 4 Å (finger type “AG”), and to interact only with γ-phosphate if only the second distance met the criteria (finger type “G”). Otherwise, no interaction with a Lys finger was presumed (finger type “None”).

For each complex, the AG site was interpreted as occupied by an Arg residue if the closest Arg residue was assigned an interaction type “NH1”, “NH1 weak”, “NH2”, “NH2 weak” or “Y-TYPE”. If the closest Lys was assigned an interaction type “AG”, the AG site was interpreted as occupied by a Lys residue (see Section 3.2.6, Section 3.2.7 and Section 3.2.8 for further details).

All other Arg or Lys residues located within 4 Å from γ-phosphate/γ-mimic were listed as present in “G-site”.

Proteins were assigned to major classes of P-loop NTPases according to membership in Pfam families. Each chain was treated separately, and only the Pfam domain included in Pfam clan CL0023 (“P-loop_NTPase”) and mapped to the sequence region corresponding to the nucleotide-binding site under consideration was used for the assignment. Since many Pfam domains describing P-loop NTPases were described before a coherent classification of P-loop NTPases was developed [7,8], some domain names may not be accurate.

***Visualization***: Structure superposition, manual distance measurements, and manual inspection and structure visualization were performed by Mol* Viewer [81] and PyMol v 2.5.0 [82].

***Scripts*** used to download and analyze the structures are available at github.com/servalli/pyploop (accessed on 22 June 2022).

## 3. Results

### 3.1. Generic Numbering of Key Amino Acid Residues for P-loop NTPases

Historically, different families of P-loop NTPases were studied by different research communities, each developing its own terminology. Therefore, no generic amino acid numbering for P-loop NTPases exists. For convenience, and by analogy with the Ballesteros–Weinstein numbering scheme for the G-protein-coupled receptors [83], we introduce here a generic residue numbering for the conserved regions of P-loop NTPases. Without this generic nomenclature, our comparison of stimulatory mechanisms in diverse classes of P-loop NTPases, as performed here and in the companion paper of Kozlova et al. [77], would hardly be possible.

First, we chose highly conserved residues as benchmark references. In the Walker A motif, this is the conserved Lys residue (K^WA^) that forms H-bonds with the O^1B^ oxygen atom of β-phosphate and O^2G^ atom of γ-phosphate (Figure 1). In the Walker B motif, we chose the conserved [Asp/Glu]^WB^ residue ([D/E]^WB^) that makes an H-bond with the last Ser/Thr residue of the Walker A motif (see Figure 1) and is involved in the coordination of the Mg^2+^ ion.

In addition to universal Walker A and Walker B motifs, we also invoked the Switch I motif that is located between the Walker A and Walker B motifs in NTPases of TRAFAC class, (from translation factor, Figure S1) [7,84]. The Switch I has only a single strictly conserved [Thr/Ser]^SwI^ residue ([T/S]^SwI^, colored yellow in Figure 1D) that can be used as a reference. In NTPases of the TRAFAC class, the side chain of this [T/S]^SwI^ residue coordinates the Mg^2+^ ion, its backbone **HN** group), forms an H-bond with γ-phosphate, and its backbone carbonyl group (hereafter **CO** group) stabilizes W_cat_ (Figure 1D).

In the following, we number the amino acids of the Walker A, Switch I, and Walker B motifs relatively to the reference residues, as shown in Figure 1. In this case, the Asn226 residue of MnmE GTPase in the K^WA^-3 position, three residues before the conserved lysine residue of the Walker A motif, which binds the K^+^ ion by its side chain and makes an H-bond with O^2G^ via its backbone **HN** group (see Figure 1A–D and Figure S2 and [70]), is denoted as Asn226^K−3^ or Asn^K−3^. Its backbone **HN** group proper is then labeled as **HN**^K−3^.

To distinguish the “macroscopic” interaction of the P-loop domain with its cognate activating partner (RNA, DNA, protein domain) from the consequent “microscopic” insertion of, say, an Arg finger into the catalytic site, we consistently refer to those elements that are poked into catalytic sites to stimulate hydrolysis as “stimulatory moieties” or “stimulators”; see, for instance, the dark-blue Arg fingers in Figure 1C or the K^+^ ion in Figure 1D. We followed Wittinghofer and colleagues, who wrote about “GAP-stimulated GTPase activity” in one of their pioneering works [85]. Furthermore, the original Latin meaning of “stimulus”—“a sharp stick used to poke cattle to get them to keep moving” (quoted from https://www.dictionary.com/browse/stimulus accessed on 7 July 2022)—nicely describes the function of Lys and Arg fingers in P-loop NTPases that routinely empower movements of cellular structures.

### 3.2. Global Computational Analysis of Stimulatory Patterns in the Whole Set of P-loop NTPase Structures with Bound Mg-NTP Complexes or Their Analogues

The ever-rising four-digit numbers of P-loop NTPase structures with ATP, GTP, and their analogues bound, as deposited in the PDB, demanded computational approaches. A search in the Protein Data Bank (PDB) at https://www.rcsb.org/ (accessed on 7 July 2022) [86,87] for proteins assigned to the entry IPR027417 “P-loop containing nucleoside triphosphate hydrolase” of the InterPro database [88] yielded as many as 1484 structure entries with 3666 catalytic sites with NTP or NTP-mimicking molecules bound (as of 11 September 2019; many of the structures contained several catalytic sites). The criteria for selection of fully fledged catalytic sites from this set and the routine of their subsequent structural analysis are described and depicted in the Methods section. After filtering, we obtained 3136 structures of catalytic sites containing complexes of Mg^2+^ ions with NTPs or NTP-like molecules, and these were subjected to further analysis. The relevant data for all these catalytic sites are presented in Supplementary Tables S1 and S2 (data as of 11 September 2019).

Based on the type of the molecule, the complexes could be sorted into four groups: 1043 sites contained native ATP/GTP molecules; 1612 sites contained bound nonhydrolyzable NTP analogues such as adenosine 5′-[β,γ-imido]triphosphate (AMP-PNP), guanosine 5′-[β,γ-imido]triphosphate (GMP-PNP), adenosine 5′-[*β*,*γ*-methylene]triphosphate (AMP-PCP), guanosine 5′-[*β*,*γ*-methylene]triphosphate (GMP-PCP), adenosine 5′-[γ-thio]triphosphate (ATP-γ-S), and guanosine 5′-[γ-thio]triphosphate (GTP-γ-S); 234 sites contained NDP:fluoride complexes mimicking the substrate state, such as NDP:BeF_3_ and NDP:AlF_3_; and 247 sites contained NDP:AlF_4_^−^ (204), NDP:MgF_3_^−^ (10), and ADP:VO_4_^3−^ (33) thought to be TS analogues [37,39,41,42,43].

#### 3.2.1. Stabilization of the O^2G^ Atom of γ-Phosphate by HN^K−3^ of the Walker A Motif

For the MnmE GTPase, we have shown elsewhere [70] that the insertion of a K^+^ ion and its simultaneous interaction with O^2A^, O^3B^, and O^3G^ atoms triggered the twist of γ-phosphate, leading to the formation of a new H-bond between the O^2G^ atom and **HN** of Asn226^K−3^ (Figure 1D and Figure S2). Generally, the position of **HN**^K−3^ in the vicinity of the O^2G^ atom (or the corresponding atom of an NTP analogue) is structurally conserved across P-loop NTPases, being determined by the highly conserved H-bond of **HN**^K−3^ with the bridging O^3B^ oxygen (Figure 1C,D).

**Figure 3:**
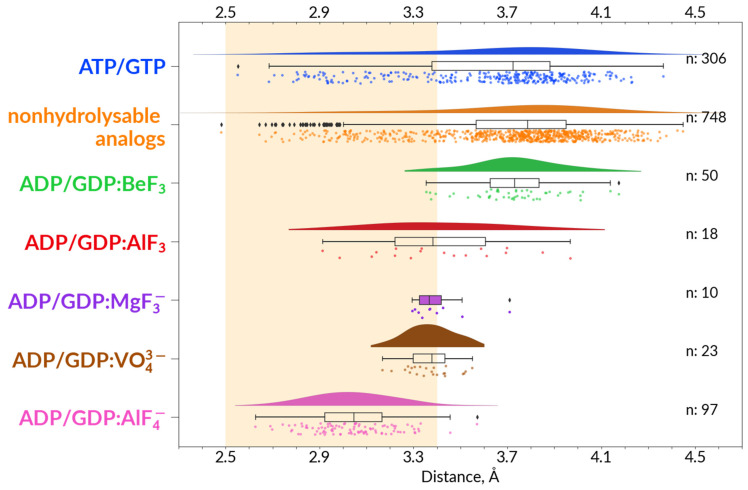
**Distances between HN^K−3^ and the O^2G^ atom or its analogue in the catalytic sites of P-loop fold NTPases.** Data are shown only for structures with resolution ≤ 2.5 Å (see Table S1 for the whole data set). For each type of complex, distances are visualized as a kernel density estimate (KDE) plot, a box plot, and individual data points, each point representing one catalytic site in one structure. For ADP/GDP:MgF_3_^−^ complexes, the density plot is not shown because of scarcity of data. The range of H-bond-compatible lengths is highlighted in amber.

To assess the possibility of a transient H-bond formation between **HN**^K−3^ and O^2G^ of γ-phosphate, as found in MD simulations of MnmE GTPases [70], we measured corresponding distances in the available structures of P-loop NTPases with bound substrates or their analogues, as described and depicted in Methods. The data obtained are presented in Table S1 and Figure 3, where the H-bond-compatible distance range is highlighted in amber. For simplicity, we used the same threshold of 3.4 Å for the H—F and H—O bonds. On the one hand, this value is somewhat lower than the threshold of 3.5 Å, as suggested for H-bonds in protein structures by Martz [89]. On the other hand, this distance corresponds to the longest F—H—N bond reported for crystalized L-cysteine-hydrogen fluoride [80]. Still, this threshold is rather arbitrary: according to Jeffrey, weak H-bonds in proteins can have donor–acceptor distances up to 4.0 Å long [90].

The data in Supplementary Table S1 show that there is a fraction with distances shorter than 3.4 Å between **HN**^K−3^ and the nearest O^2G^ atom or its structural analogue (hereafter **HN**^K−3^—O^2G^ distance) in all groups of complexes. For the ATP- and GTP-containing structures, this fraction makes up 31% of complexes (326 out of 1043 binding sites), and for the nonhydrolyzable analogues, it makes up 24% of complexes (392 out of 1612 binding sites). Among complexes with NDP:BeF_3_, the **HN**^K−3^—O^2G^ distance is <3.4 Å in 35 catalytic sites out of 171, 20%), whereas in the case of structures containing NDP:AlF_3_ this fraction makes about 28% of catalytic sites (18 out of 63).

However, the distances between **HN**^K−3^ and O^2G^ analogue are shorter than 3.4 Å in most structures with TS analogues Distances of < 3.4 Å are observed in 20 out of 33 complexes with ADP:VO_4_^3−^, in seven out of ten NDP:MgF_3_^−^ complexes, and in 163 out of 204 NDP:AlF_4_^−^ complexes (80%). 

In Figure 3 the distances between **HN**^K−3^ and O^2G^ analogue are plotted only for high-resolution structures (with resolution ≤ 2.5 Å). The data for the three TS analogues are plotted separately for clarity. In the case of this sub-set of better resolved structures, the difference with TS-like structures is even more pronounced. The fraction of structures with the **HN**^K−3^—O^2G^ distance < 3.4 Å makes up 26% (80 out of 306 binding sites) for the ATP- and GTP-containing structures, and for the nonhydrolyzable analogues, it makes up 18% of complexes (135 out of 748 binding sites). Among complexes with NDP:BeF_3_, the **HN**^K−3^—O^2G^ distance is <3.4 Å in 4 catalytic sites out of 46, whereas in the case of structures containing NDP:AlF_3_ this fraction makes up 50% of catalytic sites (9 out of 18).

However, the distances between **HN**^K−3^ and O^2G^ analogue are shorter than 3.4 Å in the vast majority of structures with bound TS analogues. Distances of < 3.4 Å are observed in 14 out of 23 complexes with ADP:VO_4_^3−^, in seven out of ten NDP:MgF_3_^−^ complexes, and in 95 out of 97 NDP:AlF_4_^−^ complexes (98%). 

Hence, the TS-like structures of catalytic sites correlate with an H-bond compatible distance between **HN**^K−3^ and O^2G^ analogue.

#### 3.2.2. Precatalytic Configurations in NTP-Containing Structures

While H-bond-compatible distances between **HN**^K−3^ and O^2G^ (or its analogue in the case of TS-like structures, Figure 3) confirmed our earlier suggestion on the importance of this H-bond for stabilization of the TS [70], we were surprised to see that the **HN**^K−3^–O^2G^ distances were H-bond compatible also in 26% of ATP- and GTP-containing structures (Figure 3, Table S1). Therefore, we manually inspected the top 100 high-resolution NTP-containing structures with shortest **HN**^K−3^–O^2G^ distances to clarify their origin.

In principle, an ATP or GTP molecule can be crystallized within an NTPase only if the latter is inactive. Not surprisingly, the ATP- or GTP-containing P-loop NTPases that were crystalized in the absence of their cognate activators were the majority in the inspected set. Although in these proteins the **HN**^K−3^–O^2G^ distances were indeed H-bond compatible, we did not explore these structures further because they could hardly help in clarifying the mechanisms of hydrolysis stimulation.

**Figure 4:**
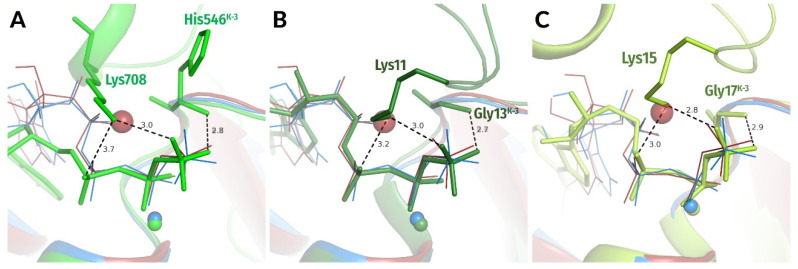
**Conformation of native ATP molecules in selected crystal structures compared to MD simulations of the MnmE GTPase.** Crystal structures of P-loop ATPases in complex with ATP molecules are shown in lime or green colors; they are superposed with conformations of GTP bound to the MnmE GTPase, as sampled from MD simulations of inactive monomer (blue) and active, K^+^-bound dimer (red); see also Figure S2 and [70]. All distances are given in ångströms. (**A**) *N*-ethylmaleimide-sensitive factor (PDB ID 1NSF, [91]). (**B**) ATPase MinD (PDB ID 3Q9L, [92]). (**C**) Chromosome segregation protein Soj (PDB ID 2BEK, [93]).

Still, we were able to identify a set of structures where NTP molecules remained not hydrolyzed despite the presence of activating partner(s). In many such complexes, the interaction of W_cat_ with γ-phosphate was hindered by the incompleteness of W_cat_ ligands, specifically caused by mutations, so that the NTP-binding sites were trapped in precatalytic configurations. Some such structures are shown in Figure 4A–C. In these enzymes, the stimulatory fingers are inserted into the catalytic sites, γ-phosphates are twisted, and the triphosphate chains are in a configuration like that observed upon the MD simulations of MnmE GTPase [70], which is superimposed as a dark-red contour (cf. Figure S2). One can see from Figure 4A–C that H-bond-compatible **HN**^K−3^-O^2G^ distances correlate with linking of O^2A^ and O^3G^ atoms by the stimulator, twist of γ-phosphate, and a more eclipsed configuration of the triphosphate chain.

#### 3.2.3. Geometry of the ADP:AlF_3_ Complex in a P-loop NTPase

It is noteworthy that a particularly flat distribution of distances between NH^K−3^ and O^2G^ analogue is observed with the AlF_3_-containing complexes (Figure 3). This flatness could be due to the anticipated presence of NDP:MgF_3_^−^ complexes in some of the structures deposited in the PDB as NDP:AlF_3_-containing structures [37,43,58]. Earlier, it was shown that, depending on pH, aluminum fluorides can make complexes with NDP in two forms, yielding NDP:AlF_4_^−^ or NDP:AlF_3_ complexes [54]. However, after identification of NDP:MgF_3_^−^ as one more TS analogue [41], Blackburn and colleagues argued that all NDP:AlF_3_ complexes from previously determined structures are, in fact, misassigned NDP:MgF_3_^−^ complexes [37,43,58]. They proposed that the low Al(OH)_3_ solubility above pH 7.5 would trigger the substitution of Al^3+^ for Mg^2+^ that is usually present in the crystallization solution of NTPases in high amounts to promote NTP binding [37,43]. Since the atomic numbers of Al and Mg atoms are very similar, specific methods, such as proton-induced X-ray emission spectroscopy (PIXE), are required to determine whether the structure contains NDP:AlF_3_ or a TS-analogue NDP:MgF_3_^−^. Application of these methods to the crystals that were studied many years ago is hardly possible.

**Figure 5:**
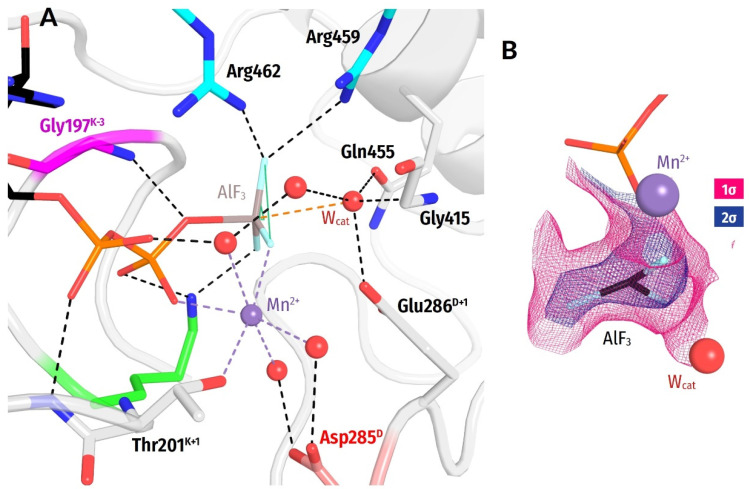
**Zika virus helicase in complex with an ADP:AlF_3_ moiety.** (**A**) Nucleotide-binding pocket of Zika virus helicase with an ADP:AlF_3_ moiety bound (PDB ID 5Y6M [2]). The Mn^2+^ ion is shown as a purple sphere; otherwise, the colour code is as in Figure 1C,D. (**B**) 2fo-fc electron density for AlF_3_ moiety contoured at 1σ and 2σ.

We manually inspected all the moieties denoted as AlF_3_ in the sampled structures of P-loop NTPases (Table S1). Upon the inspection, we identified an AlF_3_:ADP-containing structure of the Zika virus helicase with a resolution of 2 Å that is shown in Figure 5A (PDB ID 5Y6M [94]). This structure could not contain a MgF_3_^−^ ion instead of AlF_3_ because the crystallization solution contained no Mg^2+^, but only Mn^2+^ ions. No manganese fluoride complexes with more than two F^−^ ions in the Mn^2+^ coordination sphere could be found in the PDB (as of 25 June 2020). In addition, the inspection of electron density (Figure 5B) ruled out the possibility of a misassigned AlF_4_^−^.

The high resolution of this helicase structure allowed us to determine the geometry of an Mn^2+^:ADP:AlF_3_ complex in a P-loop ATPase (Figure 5). The F^3^-F^1^-F^2^-Al dihedral angle is 17.5° and the distance between the Al atom and W_cat_ is almost 3 Å compared with about 2.5 Å in MgF_3_^−^-containing complexes and about 2.0 Å in AlF_4_^−^-containing complexes [37,43]. The **HN**^K−3^—O^2G^ distance was 3.97 Å, i.e., longer than, on average, in complexes with MgF_3_^−^ and AlF_4_^−^ (cf. Figure 3). This difference is unlikely to be due to the replacement of Mg^2+^ by Mn^2+^, since the radii of the two ions are similar.

Hence, our analysis indicates that P-loop-bound NDP:AlF_3_ complexes are present in the PDB. Their AlF_3_ moiety appears to have a substrate-like geometry that differs from that of planar, TS-like MgF_3_^−^ moieties. Hence, the mixing of NDP:AlF_3_ and NDP:MgF_3_^−^ complexes might indeed contribute to the flatness of distribution of distances to **HN**^K−3^ in the case of AlF_3_-containing structures in Figure 3.

#### 3.2.4. Different Modes of AlF_4_^−^ Interaction with the Mg^2+^ Ion

**Figure 6:**
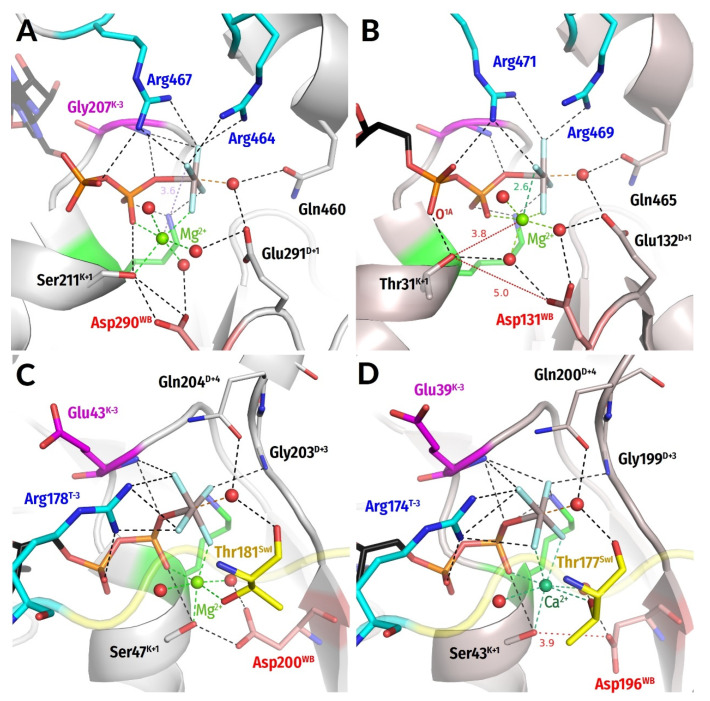
**Variation in the AlF_4_^−^ interaction with Mg^2+^ ions.** The Switch I motif is colored yellow, and the Ca^2+^ ion is shown as a teal sphere. Otherwise, the colour code is as in Figure 1C,D. The distances that are too long for H-bonds are indicated by red dotted lines. All distances are given in ångströms. (**A**) ADP:AlF_4_^−^ binding in the structure of HCV (hepatitis C virus) NS3 helicase, PDB 5E4F, chain A [96]. The Mg^2+^ ion is coordinated by the O^2B^ atom of ADP, one fluorine atom of AlF_4_^−^, the conserved Ser residue of the Walker A motif, and three water molecules. (**B**) The ADP:AlF_4_^−^ binding in the structure of double-stranded RNA (dsRNA)-dependent helicase LGP2 from chicken (PDB 5JAJ [97]). Here, AlF_4_^−^ is rotated counterclockwise and makes two bonds with the Mg^2+^ ion; the Ser^K+1^ residue is no longer directly coordinating Mg^2+^, and instead is forming an unusual bond with the O^1A^ atom of ADP. (**C**) The AlF_4_^−^ binding in the structure of guanine nucleotide-binding protein Gα_i3_ complexed with the regulator of G-protein signaling 8 (PDB 2ODE [98]). The Mg^2+^ ion is coordinated by the O^2B^ atom of GDP, one fluorine atom of AlF_4_^−^, the conserved Ser residue of Walker A motif, the conserved Thr of Switch I, and two water molecules. (**D**) In the structure of transducin Gtα complexed with Ca^2+^ instead of Mg^2+^ (PDB 1TAD [31]), AlF_4_^−^ is rotated and forms two bonds with Ca^2+^, but all interactions in the coordination sphere are preserved due to a larger ionic radius of Ca^2+^ and its ability to bind up to 8 ligands. However, the catalytic site is more open, as evidenced by a distance of 3.9 Å between Ser^K+1^ and Asp^WB^.

We also noticed the breadth of distance distributions measured in NDP:AlF_4_^−^-containing structures (Figure 3). Of course, the nucleotide-binding pockets of diverse P-loop NTPases could differ somewhat; the main classes of these enzymes, as shown in Figure S1, split even before the LUCA [7,8,13,15,21,22,23,24,25,95]. In addition, the possible artifacts of structure determination and uncertainties of such determination (as specifically addressed in Supplementary File S1) should be accounted for. Still, our examination of the NDP:AlF_4_^−^-containing structures revealed one more reason for the structural differences. It turned out that AlF_4_^−^ moieties can interact with Mg^2+^ in two different ways at least (Figure 6A–D and Supplementary Table S3 (hereafter Table S3)).

In most structures (77% of all AlF_4_^−^ complexes), only one fluorine atom interacts with Mg^2+^, like its structural counterpart, the O^1G^ atom of γ-phosphate. In this case, the next-closest fluorine atom is >3.0 Å from Mg^2+^ (Figure 6A,C and Table S3). In some structures, however, the two fluoride atoms are at similar distances of 2.0–2.7 Å from the Mg^2+^ ion and both appear to interact with it, which is only possible when the AlF_4_^−^ is rotated by approximately 45° around the O^3B^—Al bond (Figure 6B,D and Table S3). This interaction is nonphysiological because it usually prevents Mg^2+^ from interaction with one of its physiological ligands (Figure 6B and Table S3). Our manual inspection of the AlF_4_^−^ structures showed that, in general, structures with a second fluorine atom found within 3 Å from Mg^2+^ (these include structures with different degrees of AlF_4_^−^ rotation) usually have distortions in the Mg^2+^ coordination sphere, namely, missing ligands, incorrect bonding to β-phosphate, or a missing bond to Ser^K+1^. Only two catalytic sites out of 14 where two F—Mg distances are shorter than 2.7 Å appear not to have additional distortions (see Table S3). One of these two structures has a large Ca^2+^ ion instead of Mg^2+^ so that interactions in the coordination sphere are preserved due to a larger ionic radius of Ca^2+^ and its ability to bind up to 8 ligands (Figure 6D).

Accordingly, all structures with two bonds between fluoride atoms and Mg^2+^ (indicated in Table S3) appear to be suspicious as TS-state analogues because of nonphysiological coordination of the Mg^2+^ ion. This point is addressed in the Discussion section and also the companion article [77].

Still, the comparison of the same or closely related NTPases with differently bound NDP:AlF_4_^−^ complexes has proven useful. Figure 6A,B shows two structures of the Family 2 helicases (ASCE division, SF1/SF2 class, see Figure S1) with different coordination of the AlF_4_^−^ moiety. One can see that the nonphysiological coordination in Figure 6B is achieved via counterclockwise rotation of the more “physiological” configuration of the AlF_4_^−^ moiety in Figure 6A, whereby the interactions of AlF_4_^−^ with Lys^WA^ and the stimulatory Arg residues are retained in both structures. The two stimulatory fingers retain their H-bonds with AlF_4_^−^ in both configurations. This comparison shows that the residues that bind γ-phosphate appear to be adapted to the counterclockwise rotation of γ-phosphate by 30–40°. Similar counterclockwise rotation of γ-phosphate can be inferred from the comparison of G-α protein structures (kinase-GTPase division, TRAFAC class, see Figure S1) in Figure 6C,D.

The data on all found nonphysiologically bound AlF_4_^−^ moieties are highlighted in pink in Table S1 and separately summarized in Table S3.

In sum, the data in Figure 3, Figure 4 and Figure 6 indicate that the insertion of a stimulator and linking the O^2A^ and O^3G^ atoms leads to the twist of γ-phosphate and shortening of the **HN**^K−3^—O^2G^ distance in diverse NTP-containing P-loop NTPases, in support of our earlier MD simulation data [70].

#### 3.2.5. Identification of Structures with Stimulators in the Catalytic Sites

In most families of P-loop NTPases, Arg, Lys, or Asn residues serve as stimulatory moieties. We used a computational approach to inspect the patterns of their interactions with phosphate chain atoms (or their analogues) in the PDB-deposited structures of P-loop NTPases.

**Figure 7:**
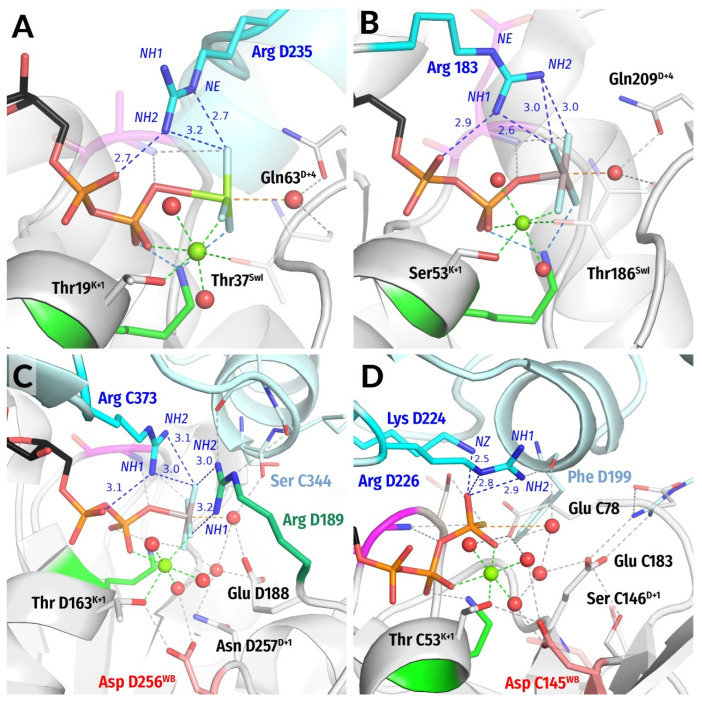
**Examples of different interaction types/stimulatory patterns involving Arg residues.** The colour code is as in Figure 1C,D. All distances are given in ångströms. (**A**) Both α- and γ-phosphates are coordinated by the NH2 atom, and an additional H-bond is formed by the NE atom (stimulatory pattern AG); the structure of the Ras-like GTPase RhoA (PDB ID 5HPY, chain B [102]) is shown. (**B**) Both α- and γ-phosphates are coordinated by the NH1 atom, and an additional H-bond is formed by the NH2 atom (stimulatory pattern AG); the structure of GTP-binding protein G(q) subunit α (PDB ID 5DO9, chain A [103]) is shown. (**C**) Both α- and γ-phosphates are coordinated by the NH1 atom, and an additional H-bond is formed by the NH2 atom (stimulatory pattern AG). An additional Arg residue provides more interactions with γ-phosphate. Notably, this residue also interacts with the backbone atom of the α-subunit that provides the main Arg finger; the structure of bovine mitochondrial F_1_-ATPase, β-subunit (PDB 1H8E, chain D [40]) is shown. (**D**) Only the γ-phosphate is coordinated by the NH2 atom of the Arg finger, while the NE atom and Lys residue provide additional H-bonds (stimulatory pattern G_multi_); the structure of circadian clock protein KaiC, (PDB 4TL8, chain C [104]) is shown.

**Figure 8:**
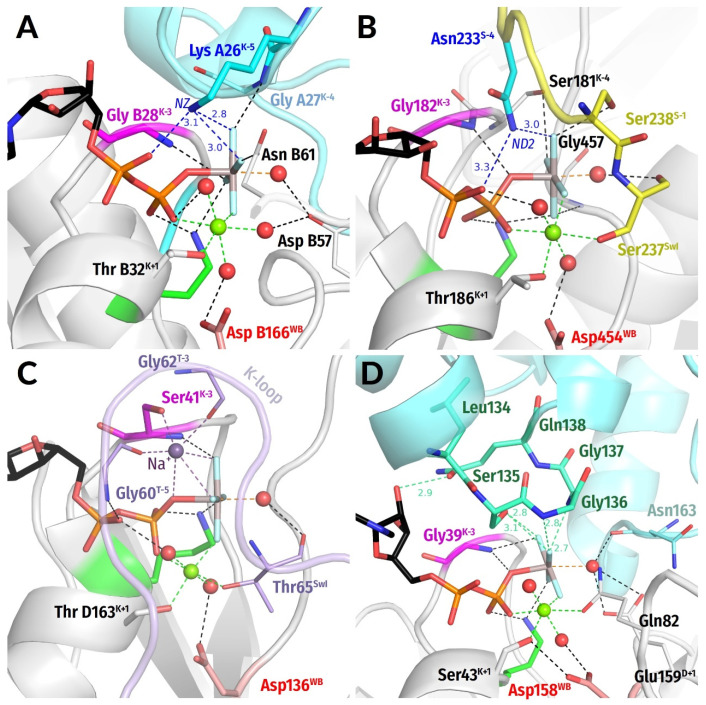
**Examples of stimulatory interactions involving moieties other than arginine residues as stimulators.** The colour code as in Figure 1C,D and Figure 7. (**A**) Interaction of a Lys finger, as provided by another subunit of the homodimer, with α- and γ-phosphates; the structure of ATPase GET3, (PDB 2WOJ, chain B [106]) is shown. (**B**) Asn finger in myosin II from ***Dictyostelium discoideum*** (PDB 1W9J). The Asn^S−4^ finger is provided by the Switch I motif, which is shown in yellow. (**C**) **A** Na^+^ ion as a stimulator in dynamin, (PDB 2X2E [107]). The switch I/K-loop motif is shown in lilac. Notably, the Gly residue coordinating Na^+^ is also contacting the O^2A^ atom of GDP. (**D**) The LSGGQ motif in a structure of ABC-ATPase MBP-maltose transporter MalK (PDB 3PUW, chain A [100]). While Ser and Gly residues are coordinating γ-phosphate via the backbone **HN** atom and the OG atom of the conserved serine residue, the Gln residue is contacting the O2′ atom of the ribose moiety in GDP molecule.

For this purpose, we analyzed, as described in the Methods section, the same 3136 PDB structures of catalytic sites that contain complexes of Mg^2+^ ions with NTPs or NTP-like molecules. For each complex, we measured the distances between oxygen atoms of the triphosphate chain (or their structural counterparts in the NTP analogues) and the amino groups of Arg, Lys, and Asn side chains within a 4 Å radius. The distances were measured towards the NE/NH1/NH2 atoms of Arg residues; the NZ atom of Lys residues; and the ND2 atom of Asn residues (see Figure 7 and Figure 8 for the atom-naming scheme). See the Methods section for further details and the scheme of the analysis pipeline. The data on all atom pairs and corresponding distances are summarized in Table S1.

We found that more than half the analyzed Mg-NTP complexes (60%) had none of the inspected residue types within the 4 Å radius around oxygen atoms. Those are structures of P-loop NTPases that were crystallized in the absence of their activating partners or are stimulated by moieties other than Asn/Arg/Lys (e.g., a monovalent cation or the signature motif of ABC-ATPases—see below).

In the remaining 1380 catalytic sites of P-loop NTPases, at least one Arg, Asn, or Lys residue (other than the reference Lys residue of the Walker A motif) was found in the proximity of the phosphate chain and categorized as a stimulator. Analysis of interactions between Arg, Lys, and Asn fingers and the phosphate chains revealed several distinct types of configurations, which hereafter are called “stimulatory patterns”. Table S2 shows how many proteins were assigned to each stimulatory pattern.

Our provisional screening of structural information on different classes of P-loop NTPases identified Arg, Lys and Asn residues, monovalent cations, and stimulatory polypeptide loops of ABC-NTPases as “main” stimulators; see [99] and the companion article [77]. Below, we describe their interaction patterns.

In addition, we quantitatively evaluated the interaction patterns of Arg, Lys, and Asn residues as main stimulators, as well as interactions of amino acid residues that serve as “auxiliary residues/stimulators.”; see the Methods section and Table S1. The interactions of monovalent cations were quantified in our previous paper [70]. For ABC-NTPases, there was not much to quantify because their only two available TS-like structures were obtained for the same protein [100]. We saw that histidine residues may serve as stimulators in several NTPase families (as discussed in Supplementary File S1 of the companion article [77]); however, for these families, there are no TS-like structures, so we cannot state this for certain. We could not identify a glutamine residue as a dedicated stimulator in any structure, although Gln residues serve as auxiliary W_cat_-coordinating ”fingers” in many P-loop NTPases (Figure 1C, Figure 4 and Figure 6 and Table S1). We address this incapacity of glutamine in the Section 4.5 of the Discussion.

#### 3.2.6. Stimulatory Patterns of Arginine Fingers

Arginine fingers are the most widespread stimulatory moieties among P-loop NTPases. In an arginine side chain, the positive charge is distributed over three nitrogen atoms of the guanidinium group. In principle, each of these atoms can interact with the phosphate chain. Consequently, we observed a variety of interactions for the Arg fingers.

In most cases, the type of stimulatory pattern was assigned automatically based on the H-bond compatibility of distances between the NTP molecule or its analogue and nearby Arg residue(s). Here, we relied on Jeffrey, who categorized H-bonds with donor–acceptor distances of 2.2–2.5 Å as “strong, mostly covalent,” those with distances of 2.5–3.2 Å as “moderate, mostly electrostatic,” and H-bonds of 3.2–4.0 Å as “weak, electrostatic” [90].

Due to inconsistencies in the atom numbering and differences among NTP analogues, we measured the distances from the Arg side-chain nitrogen atoms to the nearest oxygen atom of α-phosphate (hereafter Oα) and the nearest oxygen of γ-phosphate (hereafter Oγ) or the corresponding atom in NTP analogues. Hereafter, for simplicity, we will use “γ-phosphate” for both γ-phosphate proper and its analogues.

Several sets of criteria were applied consecutively, with each following criterion applied only to the cases that did not match any of the previous criteria:

(1) If both distances NH1-Oα and NH1-Oγ did not exceed 3.2 Å, the interaction type “NH1” was assigned, meaning that the NH1 atom forms H-bonds with both α- and γ-phosphates. Similarly, “NH2” interaction type was assigned if both distances NH2-Oα and NH2-Oγ were less than 3.2 Å.
(2) If both distances NH1-Oα and NH1-Oγ did not exceed 4 Å, whereas both distances NH2-Oα and NH2-Oγ are longer than 4 Å, the interaction type “NH1 weak” was assigned, meaning that the NH1 atom forms weak interactions with both α- and γ-phosphates. Analogous criteria were used to assign the “NH2 weak” interaction type.
(3) If at least one of the distances NH1-Oγ and NH2-Oγ did not exceed 3.2 Å, whereas both distances NH1-Oα and NH2-Oα are longer than 4 Å, the interaction type “only gamma” was assigned. Similarly, if at least one of the distances NH1-Oγ and NH2-Oγ do not exceed 4 Å, but both distances NH1-Oα and NH2-Oα are longer than 4 Å, the interaction type “Only gamma weak” was assigned.
(4) If all distances between NH1/NH2 atoms and the nearest oxygen (or fluorine) atoms of α- and γ-phosphates exceeded 4 Å, the Arg residue was considered not to be a stimulatory finger (interaction type “none”).

The remaining cases, which did not match any of these criteria, were inspected manually (see below). After the interaction types were assigned to all structures under investigation, the interaction types were attributed to particular stimulatory patterns and their frequencies were assessed (Table S3).

Arg residues in the proximity of the phosphate chain were identified as stimulatory moieties in 981 cases. A majority of Arg fingers link α- and γ-phosphates by their NH1 or NH2 groups and fall into NH1, ”NH1 weak,” NH2, and “NH2 weak” interaction types, which together are grouped into the stimulatory pattern “AG,” seen in the case of 63% of all identified Arg fingers (Tables S1 and S2, Figure 7A–C). Among the structures with TS analogues, the fraction of this stimulatory pattern reaches 94%. In contrast, in complexes with ATP or GTP molecules, only 56% of interactions could be categorized in this way.

In most remaining structures, Arg fingers show interaction types “only gamma”/”only gamma weak” and interact only with oxygen atom(s) of γ-phosphate or their analogues (Figure 7D, stimulatory pattern “G”). This stimulatory pattern was identified in 39% of complexes with ATP/GTP, 47% of complexes with nonhydrolyzable NTP analogues, and only 6% of complexes with TS analogues (Table S2).

The remaining 33 complexes, which account for 3% of all Arg fingers, did not match any of these patterns. In these 33 cases, one NH1/NH2 atom of the Arg residue forms an H-bond with α-phosphate, whereas the other NH2/NH1 atom forms another H-bond with γ-phosphate. We refer to such Y-shaped interactions as “Y-interactions” or “Y-patterns.” Since such Y-interactions are seen only in a small fraction of catalytic sites, we inspected each of these sites manually. The results of this inspection are presented in Supplementary File S1. As argued and illustrated in Supplementary File S1, there are reasons to consider all cases of Y-interactions as structure determination/crystallization artifacts of a diverse nature.

Since the guanidinium group of Arg residues can donate several H-bonds, further H-bonds are seen between amino groups of the Arg finger and the oxygen atoms of the γ-phosphate (or its mimicking group). There are two types of such additional bonds: formed by the NE atom and formed by NH1/NH2 groups not involved in the main stimulatory interaction, as exemplified by Figure 7.

The NE atoms of Arg fingers are often located at the H-bond distances from the γ-phosphate. Such interactions are documented for 10% of all Arg fingers, both for those Arg fingers that interact only with the γ-phosphate (Figure 7D) and for those fingers that coordinate both α- and γ-phosphate with the NH2 atom (Figure 7A).

An additional H-bond can also be formed by an NH1/NH2 atom that is not involved in the main stimulatory interaction. Usually, this occurs when one NH1/NH2 atom coordinates both α- and γ-phosphates. In 51% of such complexes, the other atom (NH2/NH1 correspondingly) forms an H-bond with γ-phosphate (Figure 7B,C). This interaction is particularly common in complexes with TS analogues (77%). Finally, when the Arg finger interacts only with γ-phosphate, it can accept H-bonds from both NH1 and NH2 atoms, as observed in 13% of complexes with such an interaction pattern (Table S2). In these cases, the longer H-bond was categorized as the “auxiliary” interaction. See Tables S1 and S2 for the complete data set.

Overall, the NH2/NH1 groups of Arg fingers that interact only with γ-phosphate or its analogue are often assisted—in 40% of such complexes, additional bonds are provided by the second NH1/NH2 atom, NE atom, or an additional Arg/Lys finger (Figure 7D). In this case, one can speak about the stimulatory pattern G_multi_. The Arg fingers in the AG position can also receive assistance (Figure 7C). For example, in the FtsK DNA translocase structure (PDB ID 6T8B, chain C [101]), the Arg residue interacts with α- and γ-phosphates via the NH2 atom, while the Lys finger reaches the γ-phosphate. Arg fingers reaching only γ-phosphate often contact residues involved in the coordination of W_cat_ (see Figure 7C,D).

#### 3.2.7. Stimulatory Patterns of Lysine Fingers

Lys residues were assumed to be present in an AG site if both NZ-Oα and NZ-Oγ distances were shorter than 4 Å (stimulatory pattern “AG”—see Figure 8A and Table S1) and to interact only with γ-phosphate only if the second distance met the criteria (stimulatory pattern “G” in Table S1). Otherwise, no interaction with a Lys finger was presumed (pattern “None”), see Table S1.

Lys fingers were identified in 141 structures. One typical pattern is with the NZ atom of Lys interacting with both α- and γ-phosphates, similarly to a K^+^ ion in K^+^-dependent P-loop NTPases, cf. Figure 1D and [70]. Although a Lys finger interacts with both α- and γ-phosphates in 22% of all cases (we categorize these cases as stimulatory patterns AG (Figure 8A), the fraction of such interactions was as high as 84% in complexes with TS analogues (Tables S1 and S2). When the NZ atom of Lys interacts only with the γ-phosphate (pattern “G”), another Arg residue is also often involved in the interaction with the γ-phosphate (in 78% of cases—Figure 7D). Six catalytic sites had Lys fingers coordinating both α- and γ-phosphate and an additional Arg residue in the proximity of γ-phosphate. All these sites belong to the subunits of the large T antigen (PDB ID 1SVM, in complex with ATP [105]). See Table S1 for details.

#### 3.2.8. Interaction Patterns of Asparagine Fingers

Asparagine residues were classified as stimulatory fingers when both ND2-Oα and ND2-Oγ distances were shorter than 4 Å (stimulatory pattern “AG”—see Figure 8B and Table S1). The Asn residues were found to be in contact with both α- and γ-phosphate groups in 67 complexes (Figure 8B and Table S1). All these structures belong to myosin or kinesin families (PF00063, PF00225).

More common are auxiliary Asn residues, which were found in the proximity of γ-phosphates in 248 catalytic sites, in addition to the “main” stimulators. These auxiliary Asn residues are indicated in Table S1.

#### 3.2.9. Quantitative Summary of Stimulatory Interactions of Arg, Lys, and Asn Fingers in P-loop NTPases

As summarized in Tables S1 and S2 and shown in Figure 7 and Figure 8A,B, most of the analyzed P-loop NTPase complexes with stimulatory Arg, Lys, or Asn residue(s) positioned next to the triphosphate chain (or its structural analogue) possess either a residue providing an amino group to interact with both α- and γ-phosphates (stimulatory pattern AG, 56.6% with Y-interactions), or (2) an amino-group-providing residue(s) forming multiple bonds with γ-phosphate (stimulatory pattern G_multi_, 25.6%). In the rest of the cases (17.8%), only one amino group is contacting γ-phosphate (stimulatory pattern G_lone_).

In the case of TS analogues, the fraction of catalytic sites with stimulatory pattern AG is remarkably high: all structures with ADP:VO_4_^3−^ and NDP:MgF_3_^−^ that possess any kind of stimulatory residue have it interacting with both α- and γ-phosphates. The same interaction is observed in 75% of NDP:AlF_4_^−^ complexes, whereas 22% of them display the stimulatory pattern G_multi_. Only 3% of TS analogues show stimulatory pattern G_lone_ with a single amino group contacting γ-phosphate.

The complete data on diverse kinds of auxiliary residues additionally stabilizing the negatively charged γ-phosphate and/or W_cat_ in all the studied structures are presented in Tables S1 and S2.

#### 3.2.10. Stimulation by Monovalent Cations

In at least two clades of P-loop NTPases, monovalent cations serve as stimulators, as already elucidated in a separate article [70]. First, many TRAFAC class GTPases are stimulated by K^+^ ions. The formation of a cation-binding site requires a particular positioning of the Switch I loop (dubbed K-loop in K^+^-dependent NTPases [29]), which is achieved either by the specific interaction of the P-loop domain with its activating partner(s)—protein and/or RNA molecules [70]—or can be induced by binding of a TS analogue, such as GDP:AlF_4_^−^ (Figure 1D). The K^+^ ion in MnmE GTPase is coordinated by the O^2A^, O^3B^, and O^3G^ atoms of the triphosphate chain, two **CO** groups of the K-loop, and the side chain of Asn^K−3^ (Figure 1F and Figure S2) [29,35]). In the unique eukaryotic protein family of dynamins (Figure 8C), NTP hydrolysis can be stimulated by either K^+^ or Na^+^ ions [107]. Here a Na^+^ or a K^+^ ion interacts only with the O^3B^ and O^3G^ atoms but does not reach the O^2A^ atom. See [70] for further details.

Second, in archaeal and eukaryotic RadA/Rad51-like recombinases of the RecA/F_1_ class, the positions that are taken by terminal groups of stimulatory Lys/Arg residues in other proteins of this class are occupied by two K^+^ ions, which might interact either only with γ-phosphate, or with γ-phosphate and W_cat_, as could be inferred from available structures with substrate analogues bound; see Figure 6D in the companion article [77] and [70,108,109]. No TS-like structures were available for these K^+^-dependent enzymes.

#### 3.2.11. Stimulatory Interactions in ABC-NTPases

ABC (*ATP-binding cassette*) NTPases are multidomain proteins that usually operate as homo- or heterodimers [110]. Members of the ABC class make several families named alphabetically from A to I [111]. Most of these families contain members that possess transmembrane domains and operate as genuine ATP-driven membrane transporters where the P-loop domains hydrolyze ATP. However, the members of the ABCE and ABCF families have no transmembrane domain(s) [112].

As discussed in more detail in the companion article [77], activation of ABC-transporter ATPases (ABC-ATPases) is triggered by the transported substrate and accompanied by dimerization of P-loop domains. Upon dimerization, each monomer, instead of an amino acid “finger,” inserts a whole signature motif LSGGQ into the catalytic pocket of the other monomer (Figure 8D). Some soluble ABC-NTPases have a noncanonical signature motif (e.g., CSAGQ in Rad50 [113] and xSTFx in MutS [114]). In some cases, the last residue of the motif is Glu or even Trp [115]. Thus, the serine residue is the most conserved member of the signature motif.

Two structures with ADP:VO_4_ and ADP:AlF_4_^−^ as TS analogues bound are available for the maltose transporter complex [100]. See Figure 8D here and Figure 5D in the companion article [77]. In both structures, the side chain of serine and the backbone **HN** of the second glycine residue of the signature motif (LSGGQ) interact with the O^3G^ atom of γ-phosphate. The side chain of serine is located between the α- and γ-phosphates, approximately in the position of the Na^+^ ion in dynamin-like proteins, cf. Figure 8C with Figure 8D.

## 4. Discussion

Here, we report the results of a computational comparative structural analysis of 3136 catalytic sites of P-loop NTPases with nucleoside triphosphates or their analogues bound. The aim of the analysis was to find common features in their stimulatory patterns and to use this information for elucidating the common mechanism of these enzymes.

In sum, P-loop NTPases exhibit various stimulatory patterns (Figure 1, Figure 2, Figure 3, Figure 4, Figure 5, Figure 6, Figure 7 and Figure 8 and Tables S1 and S2) involving diverse atoms of stimulators that interact with different phosphate groups and in some cases also with the W_cat_ molecules (Figure 1C).

And still, our comparative structural analysis with emphasis on TS-like structures showed that highly diverse stimulatory moieties affect the triphosphate chain in a similar way, as discussed in the sections below.

These interactions can play an instrumental role both in twisting the γ-phosphate group upon catalytic transition and, as discussed in more detail in the companion paper [77], in constricting the catalytic site and the catalytic proton transfer.

### 4.1. Stabilization of the O^2G^ Atom of γ-phosphate by **HN**^K−3^ of the Walker A Motif

For the MnmE GTPase, we have shown earlier [70] that the insertion of a K^+^ ion and its simultaneous interaction with O^2A^, O^3B^, and O^3G^ atoms triggered the twist of γ-phosphate, leading to the formation of a new H-bond between the O^2G^ atom and **HN** of Asn226^K−3^ (Figure 1D and Figure S2). We have speculated that this additional H-bond may promote hydrolysis by increasing the nucleophilicity of the P^G^ atom and making it prone to attack by OH^−^_cat_.

We measured corresponding distances in the available structures of P-loop NTPases with bound substrates or their analogues (Figure 3 and Table S1). For simplicity, we used the same threshold of 3.4 Å for the H—F and H—O bonds. Although in all group of structures there is a fraction with distances shorter than 3.4 Å between **HN**^K−3^ and the nearest O^2G^ atom or its structural analogue, this fraction is much larger in the structures with bound TS analogues reaching 98% in the case of NDP:AlF_4_^−^ complexes (resolution better than 2.5 Å). Hence, the TS-like structures of catalytic sites correlate with an H-bond-compatible distance between **HN**^K−3^ and O^2G^ or its analogue (Figure 3).

Twisted γ-phosphates and H-bond-compatible **HN**^K−3^—O^2G^ distances are also seen in diverse NTP-containing proteins that were crystalized after being trapped in their pretransition configurations in the presence of inserted stimulators (Figure 4A–C, Table S1).

Noteworthily, the H-bond between **HN**^K−3^ and O^2G^ or its analogue is formed in TS-like structures not instead of the H-bond between **HN**^K−3^ and the O^3B^ atom, but in addition to it. The interaction of **HN**^K−3^ both with the O^3B^ atom of the leaving GDP moiety and the O^2G^ atom of γ-phosphate in the TS might be important for catalysis. Both bonds would increase the electrophilicity of the P^G^ atom and make it prone to a nucleophilic attack by OH^–^_cat_. At the same time, the H-bond between **HN**^K−3^ and O^2G^ or its analogue stabilizes the twisted γ-phosphate configuration, which appears to be catalytically productive (see also below).

Although only the backbone **HN**^K−3^ is involved in the interaction with O^2G^, this position is consistently occupied by either Gly or other small (Ala or Ser) residue in most classes of P-loop NTPases [7,8,10]. The presence of small residues should enable the flexibility of the backbone, which may be important for simultaneous interaction of **HN**^K−3^ with O^3B^ and O^2G^ atoms in the TS.

Our data attribute a new and complex function to the K-3 residue of the P-loop. In human potentially oncogenic Ras GTPases, this position is occupied by a Gly13 residue. Until now, it has been unclear why the mutations of Gly13^K−3^ residue cause various types of cancer [16,17,18], even when Gly is replaced by small amino acids such as Cys or Val with their side chains directed outward from the catalytic pocket. In the scheme proposed here, the oncogenicity of Gly13Cys or Gly13Val mutants of human Ras GTPases may be due to insufficient backbone flexibility in the absence of Gly13.

### 4.2. Linking of α- and γ-Phosphates by the Stimulator

In most classes of P-loop NTPases, at least one stimulator, provided either by the same P-loop domain or by another domain/protein, gets inserted between α-and γ-phosphates, which implies the possibility of simultaneous interaction with O^2A^ and O^3G^ atoms (stimulatory pattern AG). The AG pattern was observed in 56.6% of cases.

As seen in Figure 1 and Figure 4, Figure 5, Figure 6, Figure 7 and Figure 8, the stimulator in the AG site is frequently complemented by a second auxiliary stimulator (finger) that interacts with γ-phosphate, see also Tables S1 and S2.

Simultaneous interaction of a single amino group or a K^+^ ion with O^2A^ and O^3G^ atoms is possible if the triphosphate chain bends (as observed with ATP or GTP molecules in water in the presence of Na^+^ ions [70]) or when the γ-phosphate group twists. The bending of the triphosphate chain of a P-loop-bound NTP molecule is not possible because of its multiple bonds with the protein, see Figure 1C,D, the companion article [77], and [70]. At the same time, the examination of available structures revealed cases that can be considered evidence of γ-phosphate twist in the TS (see Figure 4 and Figure 6), in support of our earlier predictions from MD simulations [70].

Quantification of the interaction types for computationally analyzed catalytic sites (Tables S1 and S2) showed that the AG stimulatory pattern dramatically prevailed in the structures containing TS analogues. This pattern was observed in all structures with ADP:VO_4_^3−^ and NDP:MgF_3_^−^and in 75% of NDP:AlF_4_^−^-containing structures. The fractions of this stimulatory pattern in structures containing ATP, GTP, or their non-hydrolyzable analogues are smaller (Table S2). In many cases, the Arg residue is in the AG site in the presence of a TS analogue but “outside” when the substrate or its analogue are bound. Apparently, catalytic sites constrict additionally in the transition state. We elaborate on this point in the companion article [77].

The notable feature is the apparent scarcity—if not complete absence—of Y-patterns with NH1 and NH2 groups of an Arg finger separately interacting with α- and γ-phosphates, respectively (see Table S1). The Y-pattern is not observed in a single structure with a bound TS analogue, and it is such structures that enable us to judge with certainty the stimulatory pattern in a particular ATPase. Our analysis has shown that the few structures with the Y-pattern are likely to be artifacts either of crystallization or of structure determination, as substantiated in Supplementary File S1.

Outside of P-loop NTPases, however, the Y-pattern of Arg interaction is very common, especially in protein–DNA complexes, where one Arg residue often donates its NH1 and NH2 groups to neighboring phosphate groups of the DNA backbone [116,117]. In the case of P-loop NTPases, however, a Y-linked Arg residue would fix the O^2A^ and O^3G^ atoms approximately 6.1 Å apart and prevent the twist of γ-phosphate. The apparent absence of Y-patterns in the examined structures of P-loop NTPases can be seen as further evidence in favor of the γ-phosphate twist as the key hydrolysis-initiating configuration change in P-loop NTPases.

Hence, linking the O^2A^ and O^3G^ atoms by the stimulator, independently of whether it is an Arg, Lys, Asn, residue or a K^+^ ion, causes a counterclockwise twist of γ-phosphate that appears to correlate with the formation of a new H-bond between the O^2G^ atom (or its counterpart in NTP analogues) and the backbone **HN**^K−3^ group of the P-loop.

### 4.3. Interaction of the Stimulator with γ-Phosphate Only

In the remaining 43.3% of P-loop NTPase structures with determined stimulatory pattern, the stimulators interact only with γ-phosphate (stimulatory pattern G). In most such structures (25.6%), γ-phosphate is involved in several interactions with distinct amino groups of an Arg finger and/or with auxiliary stimulators (stimulatory pattern G_multi_). See Figure 7D and Figure 8D and [118].

In 17.7% of structures, our computational analysis has reported only one H-bond between the stimulatory residue and γ-phosphate (stimulatory pattern G_lone_). However, in many cases, the crystal structure does not contain all the partners involved in the activation, or additional stimulatory fingers are present but are too remote because of crystallization artifacts. In fact, many catalytic sites exhibiting a G_lone_ pattern have counterparts with a richer network of H-bonds around γ-phosphate in other subunits of homooligomers of the same protein (47.6%) or in proteins belonging to the same Pfam domain (75%). Consequently, the value of 17.7% should be at least halved.

While linking of O^2A^ and O^3G^ atoms by a stimulator enforces a counterclockwise rotation of γ-phosphate, it is not clear yet what conformational changes are caused by stimulators that interact only with γ-phosphate. There is indirect evidence that γ-phosphate may be twisted clockwise in RecA NTPases, see [44,119]. In addition, in the structure of the ABC-ATPase of the maltose transporter (PDB ID 3PUW, [100]) the AlF_4_^−^ moiety is slightly twisted clockwise (Figure 8D).

Even interacting only with γ-phosphate, the stimulator is often located between α- and γ-phosphates, as in dynamins (Figure 8C) or ABC-ATPases (Figure 8D), and connected to the “head” of the NTP molecule. For instance, the Na^+^ ion in dynamins, while not reaching the α-phosphate directly, is connected to it via two noncovalent bonds (Figure 8C). The signature motif of ABC-ATPases is H-bonded via conserved Ser and Gln residues to the O2′ atom of the ribose (Figure 8D). Such connectivity may strengthen the mechanistic impact on γ-phosphate.

In sum, our structural analysis shows that in all cases where stimulators reach only γ-phosphate, these stimulators, independently on whether they are Arg or Lys residues, K^+^ or Na^+^ ions, or signature motifs of ABC-ATPases, are in position to twist or pull the γ-phosphate group.

### 4.4. Role of Mechanistic Bonding in the Common Stimulation Mechanism of P-loop NTPases

Looking together at all types of identified stimulatory patterns provides some additional clues about the mechanisms of hydrolysis stimulation. Without challenging the previously proposed tentative stimulatory effects referred to in the Introduction section, our structure analysis indicates that none of so far suggested mechanisms is common to all P-loop NTPases. Indeed, it is beyond doubt that the positive charges of Arg/Lys fingers or K^+^/Na^+^ ions, by interacting with oxygen atoms of γ-phosphate, would make the P^G^ atom more prone to the nucleophilic attack, as suggested by Warshel and colleagues [60,61,62] and as calculated by Rudack et al. [67]. The positive charge of stimulators could also compensate for the negative charge that develops at β-phosphate upon the breakaway of γ-phosphate [49,66]. Nevertheless, the absence of a positive charge on the stimulatory signature motifs of ABC ATPases (Figure 8D) does not stop them from triggering ATP hydrolysis. In addition, expelling water molecules out of the catalytic pocket by stimulatory Arg fingers may provide an entropic gain, as suggested by Kotting and collegues [63]. However, such effects are hardly to be expected when K^+^ or Na^+^ ions act as stimulators and immobilize water molecules in the catalytic pocket (Figure 1D and Figure 8C). Reorientation of the W_cat_ molecule into the attack position and its polarization, as suggested by Jin and colleagues [43,64,65], can be realized by those Arg or Lys fingers that reach W_cat_ (Figure 7D), but not by most other stimulators.

Our analysis points to the importance of mechanistic interaction of stimulators with the γ-phosphate group, which is the only feature shared by all inspected structures. The importance of this interaction is exemplified by NTPases that are stimulated by moieties with only a minute positive charge. These are ABC-NTPases, where the signature LSGG[Q/E] motif interacts with γ-phosphate via the side chain of its serine residue and the backbone **HN** of the second glycine residue, see Figure 8D and [100]. Other examples are the kinesin and myosin families, where the Asn finger inserts its NH group between α- and γ-phosphates, see Figure 8B and [120,121,122]. It is unlikely that small partial electric charges of Ser or Asn side chains could be decisive for catalysis in these cases; rather, their mechanistic H-bonding to the O^3G^ atom appears to be the key.

The mechanistic nature of the stimulatory interaction is consistent with the predominance of Arg residues as stimulators (Tables S1–S3). First, a guanidinium group could donate up to three H-bonds for interaction with the oxygens of triphosphate. Second, the strength of H-bonds between guanidinium groups and phosphate anions has been shown to be comparable to that of covalent bonds [123,124,125].

The advantage of multiple bonds for mechanistic interaction rationalizes the preference for multiple stimulatory fingers (Table S2), as well as the choice of the stimulatory signature motif by omnipresent ABC ATPases. This motif is electrically neutral but donates several H-bonds to the O^3G^ atom.

Consequently, the common denominator of stimulatory patterns in diverse P-loop NTPases is the mechanistic interaction of stimulators with the γ-phosphate group; this interaction is observed in all analyzed TS-like structures (Figure 1C,D and Figure 4, Figure 5, Figure 6, Figure 7 and Figure 8, Tables S1 and S2) and can be inferred from many other structures, specifically those that can be related to posttransition states, see the companion paper [77] and [119].

Mechanistic interaction with the γ-phosphate group may promote hydrolysis in different ways; for instance, it may destabilize the O^3B^—P^G^ bond and/or make the triphosphate chain almost fully eclipsed, and/or facilitate the inversion of γ-phosphate see the companion paper [77] and [70]. Notably, any turn of γ-phosphate inevitably disturbs the coordination sphere of Mg^2+^, since the O^1G^ atom of γ-phosphate is one of the Mg^2+^ ligands (Figure 1B–D). In P-loop NTPases, the O^1G^ atom is negatively charged, so that its displacement, by affecting the proton affinity of the other five Mg^2+^ ligands, may trigger the deprotonation of W_cat_. In more detail, we address this point in the companion article [77] where we further use comparative structure analysis to reconstruct those steps of the catalytic transition that follow the interaction with the stimulators.

### 4.5. The Puzzling Absence of Glutamine Residues as Stimulators

Glutamine residues are involved in coordination of W_cat_ in many P-loop NTPases, see, e.g., Figure 1C, Figure 4, Figure 5 and Figure 7A,B and the companion paper [77]. Still, Gln residues, unlike Asn residues (Figure 8B), could not be identified as actual stimulators in any of catalytic site structures. Notably, however, Gln residues occupy stimulator-like positions in non-catalytic sites of F_1_-ATPases (see a plethora of entries in Table S1). These sites, however, are non-functional (see also Supplementary File S1). It is tempting to speculate that the excessive, as compared to Asn, flexibility of the glutamine side chain may prevent it from twisting γ-phosphate.

### 4.6. Geometry of the AlF_3_ Moiety in the NDP:AlF_3_-Complexes

As a side result, we determined the geometry of an Mn^2+^-ADP:AlF_3_ complex in a P-loop ATPase (Figure 5) and thus offered a solution to the long-standing controversy as to whether NDP:AlF_3_ complexes could form in the catalytic site of P-loop NTPases under appropriate conditions or whether all such complexes are misassigned NDP:MgF_3_^−^ complexes, see [37,41,43,58]. The Mn^2+^-ADP:AlF_3_ complex was crystallized in the absence of Mg and therefore could not contain a MgF_3_^−^ moiety. The identified AlF_3_ moiety has substrate-like geometry similar to that of BeF_3_ moieties, unlike the planar, TS-like MgF_3_^−^ moieties. This non-planarity may help to discriminate the NDP:AlF_3_ complexes from NDP:MgF_3_^−^ complexes in earlier obtained crystal structures. Hence, it is realistic to sort out the P-loop-bound NDP:AlF_3_ complexes, as assigned in the PDB, into misassigned NDP:MgF_3_^−^ complexes and genuine NDP:AlF_3_ complexes.

### 4.7. Unwelcome Mode of AlF_4_^−^ Binding

Quite unexpectedly, we found that the AlF_4_^−^ moieties are bound in some catalytic sites in such a way that two fluorine atoms interact with the Mg^2+^ ion. In most of these cases, the coordination bond between the Mg^2+^ ion and its ligand #4, [Ser/Thr]^K+1^, is lost, leading to a distortion of the catalytic site (Figure 6, Tables S1 and S3). This finding is alarming because fluoride complexes are deservedly vaunted as powerful TS analogues, and structures containing them are commonly interpreted as TS-like [37,39,43]. Still, our data show that the integrity of Mg^2+^ coordination in the presence of NDP: AlF_4_^−^ should be evaluated separately for each enzyme structure before linking the corresponding structural data to the catalytic mechanism. Hence, AlF_4_^−^-containing P-loop NTPases can be used as reliable models of transition states, in support of earlier suggestions [37,39,43,58], provided that the “properness” of the interaction between AlF_4_^−^ and Mg^2+^ is checked in each particular case.

Notably, NDP:AlF_4_^−^ complexes are also used as TS analogues in other enzyme superfamilies [37,43]. It is important to check whether a similar distortion of catalytic sites by NDP:AlF_4_^−^ complexes could happen in enzymes other than P-loop NTPases.

Although this discovery was rather unpleasant as such, it provided some useful information. The “wrong” binding of AlF_4_^−^ was accompanied by its counterclockwise rotation compared to “properly bound” AlF_4_^−^ moieties, whereby the interactions of the fluoride atoms with Lys^WA^ and the stimulator were preserved. It could be inferred that the ligands of γ-phosphate oxygens are adapted to the twists of the γ-phosphate group.

## 5. Conclusions

Here, we performed the first, to the best of our knowledge, computational analysis of the stimulator-induced interactions of P-loop with substrate molecules or their analogues in all available structures of P-loop NTPases with fully fledged catalytic sites. After screening over 3100 available structures of catalytic sites with bound Mg-NTPs or their analogues, we found that seemingly different interactions between completely distinct stimulatory moieties (Arg/Asn/Lys residues, or K^+^/Na^+^ ions, or LSGGQ/E motifs) come down to only two stimulatory patterns. In most cases, at least one stimulator links the O^2A^ atom of α-phosphate with the O^3G^ atom of γ-phosphate, which requires a counterclockwise twist of the γ-phosphate group. Otherwise, stimulators interact only with the γ-phosphate group. In general, the only shared feature of all the identified stimulators seems to be the ability to enter a mechanistic interaction with the γ-phosphate group, which may enable its twist/rotation.

Our structural analysis strongly indicates that the counterclockwise twist of γ-phosphate correlates with the formation of a new H-bond between the **HN**^K−3^ group of the P-loop backbone and the O^2G^ atom of γ-phosphate. Specifically, H-bond-compatible distances between **HN**^K−3^ and the nearest oxygen/fluorine atom are seen in most structures with TS analogues bound (Figure 3, Table S1). Twisted γ-phosphates and H-bond compatible **HN**^K−3^—O^2G^ distances are also seen in diverse NTP-containing proteins that were crystalized, after being trapped with stimulator inserted, in their pretransition configurations (Figure 4, Table S1).

Since many P-loop NTPases are involved in potentially pathogenic processes, our results may be of medical significance. In particular, the suggested novel function of the K-3 residue puts certain demands on backbone flexibility at this position, which may explain why any mutation of Gly13^K−3^ in human Ras GTPases turns them into major oncogenes [16,17,18]. In addition, comparative analysis of Arg fingers in various P-loops of NTPases helps clarify why the Gly13Asp mutation in Ras GTPases is by far the most oncogenic [16,17,18]. Notably, the G_α_ subunits of heterotrimeric G-proteins, which are closely related to Ras GTPases [7], have a Glu residue at the K−3 position. After GTP hydrolysis, this residue interacts with the released Arg finger to form a salt bridge that locks the GDP molecule in the binding site [126]. In mutant Ras GTPases, the carboxy group of Asp13^K−3^ at the same position can interact with the stimulatory Arg789 finger (possibly forming a salt bridge with it), hinder the interaction of Arg789 with γ-phosphate, and thus prevent the cancellation of the oncogenic signal.

We provide a relevant outlook on the future studies of P-loop NTPases at the end of the second, companion paper [77], in which we summarize the whole set of our observations and suggest a novel mechanism of NTP hydrolysis common to all classes of P-loop NTPases.

Here, instead, we would like to give a flashback.

In 1998, Wittinghofer and colleagues published a seminal review with the telling title “GTPase-activating proteins: helping hands to complement an active site,” where they viewed activating molecules as hands with “fingers” [33]. In this context, the data presented here show that removing γ-phosphate from an NTP molecule resembles plucking an apple from a tree: the “fingers” seem to need to twist the γ-phosphate group before they can “rip it off”.

## Data Availability

Descriptions of each binding site are available in Table S1. Scripts used to generate and annotate the data and quickly visualize selected sites listed in Table S1 are available from https://github.com/servalli/pyploop (accessed on 22 June 2022).

## Supplementary Materials

The following supporting information can be downloaded at: https://www.mdpi.com/article/10.3390/biom12101345/s1, Figure S1. Major classes of P-loop fold NTPases. Figure S2. Molecular dynamics of the MnmE GTPase. File S1. Detailed consideration of structures with an atypical “Y-type” interaction pattern between the stimulatory Arg finger and the triphosphate chain (or its mimics in cases of NTP analogues). Table S1. Results of the computational analysis of all available structures of the P-loop proteins in complex with Mg-NTPs or their analogues. The Sheet A of the Microsoft Excel table contains the list with characteristics of all analyzed structures, together with indicated key functional residues of the Walker A and Walker B motifs, Arg, Lys, and Asn fingers, as well as distances from (1) the respective atoms of NTPs/their analogs to the K-3 residues and Arg/Lys fingers, as well as (2) from [Asp/Glu]^WB^ to [Ser/Thr]^K+1^. Each row contains the data for one catalytic site in one structure. Catalytic sites containing “properly bound” NDP:AlF_4_^−^ complexes that we deemed to be reliable TS-analogs (see Table S3 for detailed description of AlF_4_^−^ and Mg^2+^ binding in such structures) are marked with “y” or “*” in column “site rel”; they are colored green. The sites with “improperly bound” NDP:AlF_4_^−^ complexes are colored pink. All columns present in the Sheet A of the Table S1 (data) are described in the Sheet B. Table S2. Relative occurrence of activation patterns for Arg, Lys, and Asn fingers. Table S3. Coordination of the Mg^2+^ ion in the AlF_4_^−^-containing structures of P-loop NTPases. References [127,128,129,130,131,132,133,134] can be found in Supplementary materials.
