# Supplementary material for: Common Patterns of Hydrolysis Initiation in P-loop Fold Nucleoside Triphosphatases
Source: Biomolecules. 2022 Sep 22;12(10):1345. doi: 10.3390/biom12101345 (PMC9599529; doi:10.3390/biom12101345)
Supplement: Supplementary file 1 [file biomolecules-12-01345-s001.zip › Biomolecules_1832854_Supplementary_Figures.pdf]

Supplementary Figures to the manuscript "Common patterns of hydrolysis initiation in P-loop fold nucleoside triphosphatases - united in diversity" by

Maria I. Kozlova, Daria N. Shalaeva, Daria V. Dibrova, Armen Y Mulkidjanian

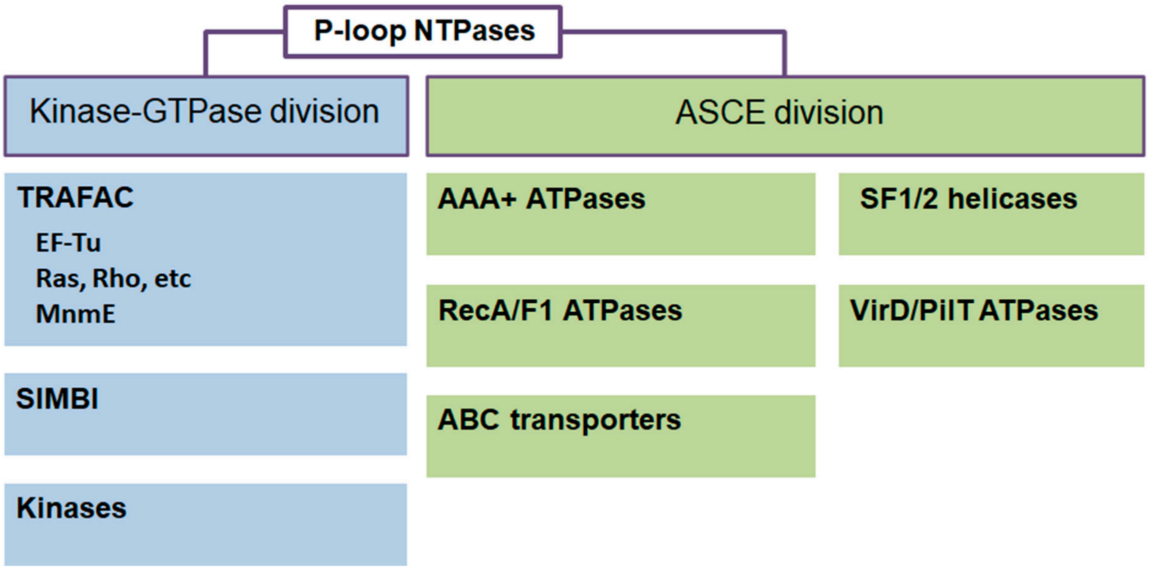

Figure S1. Major classes of P-loop fold NTPases according to [1-5].

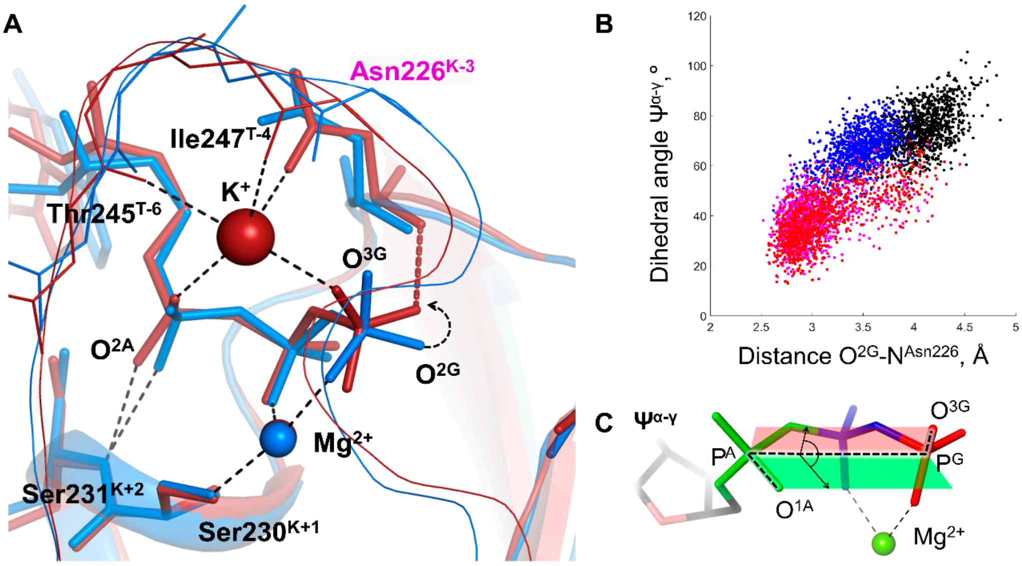

Figure S2. Molecular dynamics of the MnmE GTPase (The figure is taken from [6] and modified).

A. Superposition of the GTP-binding sites of the inactive, monomeric G-domain of MnmE (blue) and active, K^+-bound G-domain in a dimer (red); the representative structures were sampled from 100 ns simulations as described in [6]. The protein backbones are shown as cartoons; GTP and surrounding amino acid residues are shown as sticks; Mg^{2+} and K^+ ions are shown as spheres. Black dashed lines indicate hydrogen bonds and coordination bonds for cations that are present in both structures; the red dashed line indicates the H-bond between **NH$^{K\text{-}3}$** and O^{2G} that is present only in the K^+-containing dimer.

B. Conformational space of GTP in different states of MnmE GTPase. Scatter plot of the $\Psi^{\alpha\text{-}\gamma}$ dihedral angle (Y-axis) against the distance between the O^{2G} atom and **NH** of Asn226$^{K\text{-}3}$ (X-axis) as sampled from the MD simulations of three systems: (1) active dimer of G-domains with K^+ ions bound (red and magenta for individual monomers); (2) monomeric G-domain of MnmE with the K^+ ion replaced by a water molecule, blue; and (3) inactive monomer G-domain of MnmE without a full-fledged K-loop, black.

C. The dihedral angle $\Psi^{\alpha\text{-}\gamma}$ in the phosphate chain of GTP as measured for the plot on panel B.

References

[1] D.D. Leipe, Y.I. Wolf, E.V. Koonin, L. Aravind, Classification and evolution of P-loop GTPases and related ATPases, J Mol Biol 317(1) (2002) 41-72.

[2] D.D. Leipe, E.V. Koonin, L. Aravind, Evolution and classification of P-loop kinases and related proteins, J Mol Biol 333(4) (2003) 781-815.

[3] L.M. Iyer, D.D. Leipe, E.V. Koonin, L. Aravind, Evolutionary history and higher order classification of AAA+ ATPases, J. Struct. Biol. 146(1-2) (2004) 11-31.

[4] L.M. Iyer, K.S. Makarova, E.V. Koonin, L. Aravind, Comparative genomics of the FtsK-HerA superfamily of pumping ATPases: implications for the origins of chromosome segregation, cell division and viral capsid packaging, Nucleic Acids Res 32(17) (2004) 5260-79.

[5] D.D. Leipe, E.V. Koonin, L. Aravind, STAND, a class of P-loop NTPases including animal and plant regulators of programmed cell death: multiple, complex domain architectures, unusual phyletic patterns, and evolution by horizontal gene transfer, J. Mol. Biol. 343(1) (2004) 1-28.

[6] D.N. Shalaeva, D.A. Cherepanov, M.Y. Galperin, A.V. Golovin, A.Y. Mulkidjanian, Evolution of cation binding in the active sites of P-loop nucleoside triphosphatases in relation to the basic catalytic mechanism, Elife 7 (2018).
