# Supplementary material for: Common Patterns of Hydrolysis Initiation in P-loop Fold Nucleoside Triphosphatases
Source: Biomolecules. 2022 Sep 22;12(10):1345. doi: 10.3390/biom12101345 (PMC9599529; doi:10.3390/biom12101345)
Supplement: Supplementary file 1 [file biomolecules-12-01345-s001.zip › Biomolecules_1832854_Supplementary_file_1.pdf]

Supplementary File 1 to the manuscript "Common patterns of hydrolysis initiation in P-loop fold nucleoside triphosphatases - united in diversity" by

Maria I. Kozlova, Daria N. Shalaeva, Daria V. Dibrova, Armen Y Mulkidjanian

Analysis of structures with Y-like interaction pattern of arginine fingers

Our analysis revealed only 33 complexes with Y-like interaction of an Arg finger with an NTP molecule or its analog (see also Table S1). The Y-pattern is not observed in a single structure with a bound TS analog, and it is such structures that enable us to judge with certainty the stimulatory pattern in a particular ATPase. Therefore, we inspected these 33 complexes manually.

I). Seventeen of these binding sites belong to the structures obtained via electron microscopy (EM), mostly with resolution worse than 3.5 Å. Eleven of these NTP binding site structures are of catalytically inactive ATP-binding sites of α-subunits of F_1-type ATPases, from *Sus scrofa* (PDB 6J5J, chain A [1] and *Polytomella sp. Pringsheim 198.80* (all structures from [2]). In other 190 structures of non-catalytic sites, a Gln residue in K-3 position links the O^{2A} and O^{3G} atoms, see Table SF1 below. However, in these eleven structures, Gln^{K-3} does not reach the O^{2A} atom so that the "Y-type" Arg residue of the adjoining monomer takes the canonical "finger position" and enters a Y-interaction with O^{2A} and O^{3G}. However, the high-resolution X-ray structures of the same non-catalytic sites reveal the amino group of the Gln^{K-3} residue in the AG position, as discussed in the main text. We do not know why these eleven structures show a different interaction than the rest 190 structures of noncatalytic sites. Anyhow, both the Gln "plug" in 190 structures, as well as the Y-interacting Arg in eleven deviating structures are fully compatible with the major task of non-catalytic ATP binding sites, which is <u>not</u> to catalyze ATP hydrolysis.

The remaining six EM-derived complexes with Y-type interaction come from structures of oligomeric complexes where other subunits display a typical stimulatory interaction via a single NH_2 group. Only one of these structures has a resolution better than 3.5 Å.

II). We manually inspected the remaining **15** binding sites with Y-pattern (as obtained by X-ray crystallography of **13** crystal structures) and found out that they can be attributed at least to one of the following five cases (see details for each binding site in Table SF1):

1) **Presence of other complexes of the same protein with other, more common stimulatory patterns, with only one NH_2 group of an Arg finger interacting with both α- and γ-phosphates or stimulators interacting with only γ-phosphate**. Table SF1 contains five oligomeric structures where one protein subunit exhibits Y-like interaction of the Arg finger, whereas other subunits of the same protein have a different configuration of the catalytic site. No structures display two or more catalytic sites with a Y-like interaction for the same oligomeric protein.

   For some of the proteins with Y-pattern in Table SF1, there are many other structures either from the same (ten complexes) or different organisms (six complexes) that display a different binding pattern. For instance, the Y-pattern is seen in one catalytic site of the rotary ATP-synthase (PDB 3OEE, chain L). This binding pattern is not displayed either in the other catalytic sites of the same structure, or in 69 other catalytic sites from β -subunits with either ATP or its analogs bound and an Arg finger present (see Table SF1).

2) **Arg finger residue is listed as an outlier in wwPDB structure quality assessment reports.**

   In five cases, the Arg finger residues involved in Y-interactions were reported as outliers in regard to side chain geometry. Four of them are reported to possess a non-rotameric sidechain, while one residue is a bond angle outlier (NE-CZ-NH2 angle), see Table SF1.

The reports also list too-close contacts; eight sites possess an Arg finger engaged in interatomic clashes either with other amino acid residues or with atoms of nucleotide moiety that are not expected to form H-bonds. In the structure of Guanine nucleotide-binding protein G (PDB 3FFA [3]) the sidechain atoms of Arg178 are even clashing with neighboring atoms of the same residue.

Specifically, this set of cases contains the stimulatory Arg789 residue of H-RasGAP that is Y-linked with α- and γ-phosphates in the first prototypical structure of the H-Ras/RasGAP complex (PDB ID 1WQ1 [4]), which has been widely used for MD and QM/MM modeling. The PDB X-ray Structure Validation Report for this structure (accessible at https://www.rcsb.org/structure/1WQ1) indicates that the conformation of the side chain of the Arg789 finger contains at least one outlier for two of the geometric quality criteria; it is noted also that Arg789 has a non-rotameric side chain conformation, which may point to a crystallization artefact.

Notably, in earlier reported structures of highly homologous Gα-proteins the Arg finger interacted with α- and γ-phosphates via a single NH_2 group (PDB ID 1GFI, 1GIL [5]). Also in the all subsequently obtained structures of Ras-like GTPases crystallized with TS-analogs and cognate activators (with resolution < 2.0 Å, see, for instance, the high-resolution structures with PDB ID 1OW3 [6], 1TX4 [7], 3MSX, 5IRC [8]), only one NH2 group interacts both with α- and γ-phosphates. This prompts the suggestion that the Arg residue in the very first structure of the Ras/RasGAP complex (PDB ID 1WQ1 [4]) should have the same orientation. This suggestion is corroborated by MD simulations of the Ras/RasGAP which, after starting from the crystal structure, promptly yielded a conformation where a single NH_2 group interacted with α- and γ- phosphates, see e.g. [9].

3) **The distances between the NH2 group and O^{2A}, O^{3G} atoms that are too short for an H-bond (O..H-N distance less than 2.4 Å) are observed in five complexes, see Table SF1**.
4) **Electron densities (ED) are inconsistent with the positioning of the residue side chains.** We have manually evaluated electron density maps (2Fo-Fc) for all 15 sites, see examples in Fig SF1A-F. Four Arg fingers lack ED entirely (example: Fig SF1A, F), while in one site the sidechain is poorly fitted to the available density (Fig SF1E). In two cases electron density distribution is more consistent with the same NH_2 group bonded both to α- and γ-phosphates (Fig SF1E, B). Six complexes display some electron density, but it is poorly resolved in the terminal region of Arg residue, thus not allowing determination of the exact location of the guanidinium group (examples: Fig SF1C, D).
5) **Optimized structures in PDB REDO depict a different configuration of the stimulator.** We also checked the structures with Y-pattern in the PDB REDO structure databank, which contains automatically optimizes crystallographic structure models [10]. In three cases, the Y-pattern is absent from the re-refined structure, in two other sites the Arg is still inserted in a Y-like manner, however, the distance between the respective NH_2 group and the more distant phosphate group is shortened. It is worth noting that sidechains lacking electron density cannot be expected to shift considerably in a re-refined structure.

Finally, eight complexes with Y-like patterns belong to SF1/SF2 helicases (see Fig 1C in the main text for an example of a typical binding site in a SF1 helicase). Remarkably, most (105) other complexes of this class, including all the complexes with TS analogs bound, depict a single NH_2 group interacting with α- and γ-phosphates, and in seven remaining cases Arg/Lys residue(s) contacts only γ-phosphate. Almost all these complexes harbor a second Arg residue

contacting the γ-phosphate moiety. We would suggest that the catalytic site is not properly arranged in the absence of TS analog in these eight structures with Y pattern.

In sum, these findings indicate that a Y-like stimulatory pattern is unlikely to be inherent to P-loop fold NTPases; its presence in a few experimental structures might be due to poor resolution of particular residues or crystallization artefacts.

References

[1] J. Gu, L. Zhang, S. Zong, R. Guo, T. Liu, J. Yi, P. Wang, W. Zhuo, M. Yang, Cryo-EM structure of the mammalian ATP synthase tetramer bound with inhibitory protein IF1, Science 364(6445) (2019) 1068-1075.
[2] B.J. Murphy, N. Klusch, J. Langer, D.J. Mills, O. Yildiz, W. Kuhlbrandt, Rotary substates of mitochondrial ATP synthase reveal the basis of flexible F1-Fo coupling, Science 364(6446) (2019).
[3] N. Kapoor, S.T. Menon, R. Chauhan, P. Sachdev, T.P. Sakmar, Structural evidence for a sequential release mechanism for activation of heterotrimeric G proteins, J Mol Biol 393(4) (2009) 882-97.
[4] K. Scheffzek, M.R. Ahmadian, W. Kabsch, L. Wiesmuller, A. Lautwein, F. Schmitz, A. Wittinghofer, The Ras-RasGAP complex: structural basis for GTPase activation and its loss in oncogenic Ras mutants, Science 277(5324) (1997) 333-8.
[5] D.E. Coleman, A.M. Berghuis, E. Lee, M.E. Linder, A.G. Gilman, S.R. Sprang, Structures of active conformations of Gi alpha 1 and the mechanism of GTP hydrolysis, Science 265(5177) (1994) 1405-12.
[6] D.L. Graham, P.N. Lowe, G.W. Grime, M. Marsh, K. Rittinger, S.J. Smerdon, S.J. Gamblin, J.F. Eccleston, MgF(3)(-) as a transition state analog of phosphoryl transfer, Chem Biol 9(3) (2002) 375-81.
[7] K. Rittinger, P.A. Walker, J.F. Eccleston, S.J. Smerdon, S.J. Gamblin, Structure at 1.65 A of RhoA and its GTPase-activating protein in complex with a transition-state analogue, Nature 389(6652) (1997) 758-62.
[8] E. Amin, M. Jaiswal, U. Derewenda, K. Reis, K. Nouri, K.T. Koessmeier, P. Aspenstrom, A.V. Somlyo, R. Dvorsky, M.R. Ahmadian, Deciphering the Molecular and Functional Basis of RHOGAP Family Proteins: A SYSTEMATIC APPROACH TOWARD SELECTIVE INACTIVATION OF RHO FAMILY PROTEINS, J Biol Chem 291(39) (2016) 20353-71.
[9] H. Resat, T.P. Straatsma, D.A. Dixon, J.H. Miller, The arginine finger of RasGAP helps Gln-61 align the nucleophilic water in GAP-stimulated hydrolysis of GTP, Proc. Natl. Acad. Sci. USA 98(11) (2001) 6033-8.
[10] R.P. Joosten, J. Salzemann, V. Bloch, H. Stockinger, A.C. Berglund, C. Blanchet, E. Bongcam-Rudloff, C. Combet, A.L. Da Costa, G. Deleage, M. Diarena, R. Fabbretti, G. Fettahi, V. Flegel, A. Gisel, V. Kasam, T. Kervinen, E. Korpelainen, K. Mattila, M. Pagni, M. Reichstadt, V. Breton, I.J. Tickle, G. Vriend, PDB_REDO: automated re-refinement of X-ray structure models in the PDB, J Appl Crystallogr 42(Pt 3) (2009) 376-384.

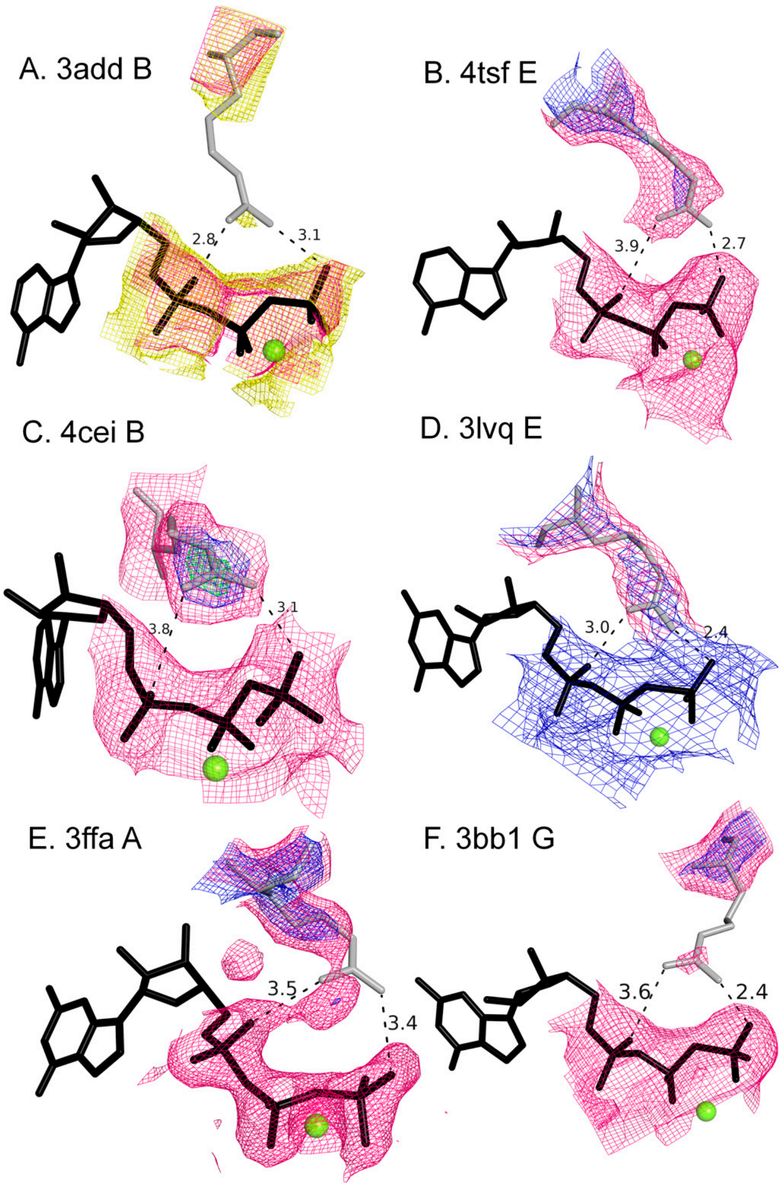

Figure SF1. 2Fo-Fc electron density maps for Arg residues contacting α- and γ-phosphate groups in a Y-like mode.

Nucleotides and their analogs are shown as black sticks, interacting Arg residues as gray sticks. Mg^{2+} ions are shown as green spheres. Density map is colored according to the contouring level (1σ in pink , 2σ in blue, 3σ in green, 0.5σ in yellow)

A. PDB 3ADD, chain B, Arg 116. Density contoured at 0.5σ and 1σ shown for phosphate chain and Arg residue.

B. PDB 4TSF, Arg E356. Electron density shown for phosphate chain is contoured at 1σ and at 1σ and 2σ for Arg residue.

C. PDB 4CEI B 283. Electron density shown for phosphate chain is contoured at 1σ and at 1σ, 2σ, 3σ for Arg residue.

D. PDB 3LVD, Arg E469. Electron density shown for phosphate chain is contoured at 2σ and at 1σ and 2σ for Arg residue.

E. PDB 3FFA, Arg A178. Density shown as in (B)

F. PDB 3BB1, Arg G133. Density shown as in (B)

Table SF1. Arginine residues contacting phosphate chain in a Y-like mode

Class	Protein name	PDB ID	Resolution, A	Nucleotide	Arg finger		Nucleotide-binding pockets in other structures of the same protein			PDB-REDO	Structure quality, wwPDB		ED evaluation	Distances
					Residue ID	Additional	Other subunit /copy***	Other structures , same organism***	Other structures, different organisms ***,#		Sidechain*	interatomic clashes **		
TRAFAC	Guanylate-binding protein 1	**2bc9**	2,8	GNP-A593	A48	_	_	**NH2** (2b92: A);<br>**NH2 weak** (2b92: B)	_	_	NR		_	Ultra-short distance to O1G (2.3 Å)
Kinases	L-seryl-tRNA(Sec) kinase	**3add**	2,4	ANP-B2002	B116	_	**NH2 weak** (A)	**NH1 weak** (3adb);<br>**NH2 weak** (3a4l, 3adc AB)	_	_	_	**3:**<br>T-N_SUG,<br>T-N_bridgN,<br>SideCh-AAB	No density	
TRAFAC	Translocase of chloroplast 34	**3bb1**	2,8	GNP-H281	G133	_	**NH2 weak** (A,E);<br>**NH1 weak** (C);<br>**ONLY GAMMA** (B,D,F,H)	_	_	**NH2:**<br>O2A - 3.82Å,<br>O1G- 2.84Å	_		Almost no density	Ultra-short distance to O1G (2.36 Å)
SF1_SF2	Eukaryotic initiation factor 4A-III	**3ex7**	2,3	ADP-AF3-C414	C370	C367: NH2-F1 (2.63)	**NH2 weak** (H)	**NH2** (2hyi CI),<br>**NH2 weak** (2j0q AB, 2j0s A, 2xb2 AX)	_	_	_		_	
TRAFAC	Guanine nucleotide-binding protein G	**3ffa**	2,3	GSP-A1	A178	_	_	**NH1** (1agr AD, 1gfi, 1shz AD, 1svk, 3d7m);<br>**NH1 weak** (1bh2);<br>**NH2 weak** (2zjy);<br>**ONLY GAMMA** (4n0d)	**<u>HUMAN:</u>**<br>**NH1**<br>(2g83 A, 2gtp AB; 2ik8 AC);<br>**NH1 weak**<br>(2g83 B)	**NH1:**<br>O1A 3.58,<br>O3G 3.44;<br>**NH2** additional:<br>O3G 3.27	NR	**12:** Internal(5), T-AAS(7)	Bad fit, by density: **NH1** bonding alpha and gamma	
TRAFAC	ADP-ribosylation factor 6 (fusion with Arf-GAP)	**3lvq**	3,38	GDP-AF3-E682	E469	_	_	**NH2** (5jcp);<br>**NH2 weak** (3lvr)	**<u>MOUSE:</u>**<br>**NH2 weak:**<br>(3bh6, 3bh7)	AF3 floated away (before: O3B-Al: 2 Å, after: 2.42Å),	_		Uncertain group position	Ultra-short distance to F3 (2.36 Å)

										both distances are longer				
RecA_F1	ATP synthase subunit beta, mitochondrial	**3oee**	2,74	ANP-M600	L375	M190 NH2-O3G (2.58)	**NH1** (X) **NH1 weak** (B,C,K,U375)	**NH1** (2hld BTU, 2xok B, 3oe7 T, 3oeh T, 3ofn L, 6b8h_BW) **NH1 weak** (2hld CKL, 2xok C, 3oe7 BCKLU, 3oeh BCKL, 3ofn BKT, 6b8h CX) **ONLY GAMMA** (3ofn V, 3oeh U)	**BOVIN:** **NH1** (1cow B, 1e1r C, 1h8e C, 1h8h B, 1nbm B, 1ohh AC, 1w0jC, 2jdi C, 2jj2 I, 2xnd C, 4yxw BC); **NH1 weak** (1bmf B, 1e1q B, 1e1r B, 1efr B, 1h8e B, 1ohh BC, 1w0j B, 2ck3 B, 2jdi B, 2jiz BI, 2jj1 BI, 2jj2 B, 2xnd B, 2wss K); **PARDP:** **NH1 weak** (5dn6 C); **ONLY GAMMA** (5dn6 F)	both distances are longer	_	**4:** T-AAS, T-N_SUG(3)	Uncertain group position	
SF1_SF2	ATP-dependent helicase/nuclease subunit A	**4cei**	2,8	ANP-A2233	A479	A873 NH2-O3G (2.81)	_	**NH1 weak**: 4cej B	_	_	NR		Uncertain group position	
SF1_SF2	ATP-dependent helicase/deoxyribonuclease subunit B	**4cei**	2,8	ANP-B2161	B283	_	_	_	_	_	NR	**1:** T-N_SUG	Uncertain group position	
SF1_SF2	ATP-dependent helicase/nuclease subunit A	**4cej**	3	ANP-A2233	A479	A873 NH2-O3G (3.13)	_	_	_	**NH2:** O2A 3.70, O3G 3.26; **NH1** additional O3G 3.16	_	**4:** T-N_bridgN (2), SideCh-AAS (2)	Uncertain group position	
RecA_F1	ATP synthase subunit alpha, mitochondrial **(see section I on EM structures)**	**4tsf**	3,2	ATP-B600	E356	_	**ONLY GAMMA** WEAK (F); NONEweak (D)	**NH2 weak** (4tt3, 4z1m) **ONLY GAMMA** (2jj2 EL, 2v7q) **ONLY GAMMA WEAK** (1e1q, 1e1r, 1nbm, 2ck3, 2jdi, 2jiz EL, 2jj1 EL, 2wss EN, 2xnd, 4tt3, 4yxw)	**ECOLI**: **NH2 weak** 3oaa cEMU **ONLY GAMMA WEAK** 3oaa bDdLT	**NH2 – O2A** shortened to 4.04 Å (previously 5.10 Å)	_		NH2 with no density, by density: same atom bonding both alpha and gamma	
SF1_SF2	Zika virus NS3 helicase	**5gjc**	2,2	ATP-A702	A462	A459 NH2-O1G (3.52)	_	**NH2** (5k8i, 5k8t, 5y4z)	**DEN4T:** **NH1 weak** (2ljr, 2ljv AB), **HCVCO:**	**NH1 – O1G** shortened to 3.81 Å from 4.41 Å	_	**1:** SideCh-N_SUG	Uncertain group position	Very short distance to O1G (2.39 Å)

									**NH2**<br>(5e4f B)<br>**NH2 weak**<br>(5e4f A,3kql AB,3kqn,3kqu ABCDE)<br>**<u>9HEPC:</u>**<br>**NH2 weak**<br>(3o8d AB, 3o8r)					
SF1_SF2	Pre-mRNA-splicing factor ATP-dependent RNA helicase PRP43	**5i8q**	4,2	ANP-A802	A430	A427 NH1-O1G (3.20)	_	_	_	_	_	**2:**<br>T-N_bridgN, T-AAS	No density	
SF1_SF2	Pre-mRNA-splicing factor ATP-dependent RNA helicase PRP43	**5i8q**	4,2	ANP-B802	B430	B427 NH1-O1G (2.91)	_	_	_	_	_	**2:**<br>T-N_bridgN, T-AAS	No density	
SF1_SF2	Zika virus helicase	**5y6m**	2	ADP-AF3-A702	A462	A459 NH2-F3 (2.96)	_	**NH2**<br>(5k8i, 5k8t, 5y4z)	**<u>DEN4T:</u>**<br>**NH1 weak** (2ljr, 2ljv AB),<br>**<u>HCVCO</u>**:<br>**NH2**<br>(5e4f B),<br>**NH2 weak**<br>(5e4f A,3kql AB,3kqn,3kqu ABCDE),<br>**<u>9HEPC:</u>**<br>**NH2 weak**<br>(3o8d AB, 3o8r)	_	bond angle outlier (NE-CZ-NH2)		_	Ultra-short distance to F3(2.22 Å); O3A is a bridging atom, shortest distance to non-bridging is 4Å

*NR – non-rotameric

** Types of interatomic clashes:

- Internal: contacts within Arg sidechain

Clashes of terminal atoms:

- T-AAS: contacts of Arg guanidinium group with sidechain of other amino acid residue
- T-N_SUG: contacts of Arg guanidinium group with ribose group of nucleotide
- T-N_bridgN: contact of one of the terminal amino groups with NH group replacing ester O3B atom in non-hydrolazable analogs ANP and GNP

Clashes of atoms other than NH1, NH2 and corresponding H atoms:

- SideCh-AAB: contacts of Arg sidechain with backbone atoms of other amino acid residue
- SideCh-AAS: contacts of Arg sidechain with sidechain of other amino acid residue
- SideCh-N_SUG: contacts of Arg sidechain with ribose group of nucleotide

*** only for corresponding Arg residues

Organism names given as Uniprot organism mnemonics: BOVIN - *Bos taurus*, ECOLI - *Escherichia coli* (strain K12), PARDP - *Paracoccus denitrificans* (strain Pd 1222), DEN4T - Dengue virus type 4 (strain Thailand/0348/1991), HCVCO - Hepatitis C virus genotype 1b (isolate Con1), 9HEPC - Hepacivirus C (unspecified), MOUSE - *Mus musculus*.
